# Comprehensive *In Vitro* Toxicity Testing of a Panel of Representative Oxide Nanomaterials: First Steps towards an Intelligent Testing Strategy

**DOI:** 10.1371/journal.pone.0127174

**Published:** 2015-05-21

**Authors:** Lucian Farcal, Fernando Torres Andón, Luisana Di Cristo, Bianca Maria Rotoli, Ovidio Bussolati, Enrico Bergamaschi, Agnieszka Mech, Nanna B. Hartmann, Kirsten Rasmussen, Juan Riego-Sintes, Jessica Ponti, Agnieszka Kinsner-Ovaskainen, François Rossi, Agnes Oomen, Peter Bos, Rui Chen, Ru Bai, Chunying Chen, Louise Rocks, Norma Fulton, Bryony Ross, Gary Hutchison, Lang Tran, Sarah Mues, Rainer Ossig, Jürgen Schnekenburger, Luisa Campagnolo, Lucia Vecchione, Antonio Pietroiusti, Bengt Fadeel

**Affiliations:** 1 Division of Molecular Toxicology, Institute of Environmental Medicine, Karolinska Institutet, Stockholm, Sweden; 2 Department of Clinical and Experimental Medicine, University of Parma, Parma, Italy; 3 Department of Biomedical, Biotechnological and Translational Sciences, University of Parma, Parma, Italy; 4 Nanobiosciences Unit, Institute for Health and Consumer Protection, European Commission—Joint Research Centre, Ispra, Italy; 5 Technical University of Denmark, Kongens Lyngby, Denmark; 6 National Institute for Public Health and the Environment, Bilthoven, The Netherlands; 7 Chinese Academy of Sciences Key Laboratory for Biomedical Effects of Nanomaterials and Nanosafety, National Center for Nanoscience & Technology of China, Beijing, P. R. China; 8 Centre for BioNano Interactions, School of Chemistry and Chemical Biology, University College Dublin, Belfield, Dublin, Ireland; 9 Centre for Nano Safety, Edinburgh Napier University, Edinburgh, United Kingdom; 10 Institute of Occupational Medicine, Edinburgh, United Kingdom; 11 Biomedizinisches Technologiezentrum, Westfälische Wilhelms-Universität, Münster, Germany; 12 Department of Biomedicine and Prevention, University of Rome Tor Vergata, Rome, Italy; North Carolina A&T State University, UNITED STATES

## Abstract

Nanomaterials (NMs) display many unique and useful physico-chemical properties. However, reliable approaches are needed for risk assessment of NMs. The present study was performed in the FP7-MARINA project, with the objective to identify and evaluate *in vitro* test methods for toxicity assessment in order to facilitate the development of an intelligent testing strategy (ITS). Six representative oxide NMs provided by the EC-JRC Nanomaterials Repository were tested in nine laboratories. The *in vitro* toxicity of NMs was evaluated in 12 cellular models representing 6 different target organs/systems (immune system, respiratory system, gastrointestinal system, reproductive organs, kidney and embryonic tissues). The toxicity assessment was conducted using 10 different assays for cytotoxicity, embryotoxicity, epithelial integrity, cytokine secretion and oxidative stress. Thorough physico-chemical characterization was performed for all tested NMs. Commercially relevant NMs with different physico-chemical properties were selected: two TiO_2_ NMs with different surface chemistry – hydrophilic (NM-103) and hydrophobic (NM-104), two forms of ZnO – uncoated (NM-110) and coated with triethoxycapryl silane (NM-111) and two SiO_2_ NMs produced by two different manufacturing techniques – precipitated (NM-200) and pyrogenic (NM-203). Cell specific toxicity effects of all NMs were observed; macrophages were the most sensitive cell type after short-term exposures (24-72h) (ZnO>SiO_2_>TiO_2_). Longer term exposure (7 to 21 days) significantly affected the cell barrier integrity in the presence of ZnO, but not TiO_2_ and SiO_2_, while the embryonic stem cell test (EST) classified the TiO_2_ NMs as potentially ‘weak-embryotoxic’ and ZnO and SiO_2_ NMs as ‘non-embryotoxic’. A hazard ranking could be established for the representative NMs tested (ZnO NM-110 > ZnO NM-111 > SiO_2_ NM-203 > SiO_2_ NM-200 > TiO_2_ NM-104 > TiO_2_ NM-103). This ranking was different in the case of embryonic tissues, for which TiO_2_ displayed higher toxicity compared with ZnO and SiO_2_. Importantly, the *in vitro* methodology applied could identify cell- and NM-specific responses, with a low variability observed between different test assays. Overall, this testing approach, based on a battery of cellular systems and test assays, complemented by an exhaustive physico-chemical characterization of NMs, could be deployed for the development of an ITS suitable for risk assessment of NMs. This study also provides a rich source of data for modeling of NM effects.

## Introduction

Due to their unique physico-chemical properties, nanomaterials (NMs) are commonly used in various applications in the industrial, electrical, pharmaceutical and biomedical fields [1] and are included in several consumer products such as cosmetics and food, or specially designed for imaging and drug delivery applications. An important mechanism involved in NM toxicity is the oxidative stress, i.e. reactive oxygen species (ROS) generation, which triggers inflammation, DNA damage, protein denaturation or lipid peroxidation [2, 3]. These biological effects can be influenced by the physico-chemical properties of the NMs (i.e. size, surface area, shape, surface chemistry, functionalization, solubility, etc.) [3–5]. As such, if a large number of variables that may determine the biological impact have to be considered, each NM would have to be evaluated individually regarding hazardous and physico-chemical properties. Therefore the development of an intelligent testing strategy (ITS) to allow risk evaluation of NMs is necessary [6]. In an ITS, data from *i) in vivo*, *ii) in vitro* tests, *iii) in silico* models and *iv)* physico-chemical properties are integrated as efficiently as possible with regard to costs, the number of experimental animals and time in order to reach a conclusion on potential risks in a specific exposure scenario [7]. In this aim, *in vitro* tests are especially relevant in an early phase of an ITS for screening purposes and for steering decisions for the choice of subsequent steps. *In vitro* tests can be used both for identification of potential, relevant toxicity endpoints as well as providing insight in the biokinetics of a specific NM.

Currently, the common approach for assessing the toxicity of NMs includes one or more cellular assays combined with rodent exposures. The *in vitro* outcomes frequently investigated include cytotoxicity, apoptosis, ROS and cytokine production and genotoxicity [8]. Moreover, the physico-chemical properties of NMs, including primary particle size, size distribution, composition, surface chemistry, shape, specific surface area, zeta potential, crystallinity, crystalline size, dissolution, solubility and redox potential [9] should be also considered when the risk assessment is performed, as these properties have been associated with their potential toxicity. Other aspects, such as the agglomeration and aggregation, stability, protein bio-corona, dosimetry or the biokinetics of the tested NMs [10] are recognized complexities that have to be taken into account when deciding if the results from *in vitro* tests are reliable, valid and useful for NMs hazard assessment. In addition, for the purpose of risk assessment, not only the test itself but, most likely, also the way it is performed may have limitations. Thus, the experimental design may need further optimization. So far, there are no standardized *in vitro* tests and experimental protocols suitable for NMs toxicity testing nor any guidelines for the extrapolation of the *in vitro* results to human health effects [11]. Therefore, the efforts should concentrate on optimizing and validating relevant and reliable *in vitro* test methods that could be used for NMs risk assessment. The essential criteria to produce robust, reliable and verified data from *in vitro* nanotoxicology tests include detailed material characterization (including physico-chemical properties before, during and after testing), use of comparable and comprehensive dose metrics and test conditions for *in vitro* assays, implementation of internal performance controls, use of reference NMs allowing comparisons between studies, performance of at least two independent methods per endpoint and implementation of nano-related interference controls. Further material characterization in the relevant biological matrix is also needed and developing methods to do this is important [12, 13]. To this end, it is important to design a battery of reliable test methods.

In order to resolve some of these challenges, the European Commission (EC) has invested considerable resources in nanosafety-related research projects; close to fifty projects are either completed or ongoing and represent a total investment of €137 million from the ‘Nanosciences, nanotechnologies, materials & new production technologies’ (NMP) and other programmes, with 13 projects (€31 million) under Framework Programme 6 (FP6) and 34 projects (€106 million) to date under FP7 [14, 15]. Thus, a considerable amount of data on the potential hazard of NMs has accrued; however, these data do not allow for general conclusions (i.e. on the applicability of the test, hazard grouping/ranking of NMs) partially due to the lack of standardized methods and reference NMs for toxicity assessment [16]. One of the projects funded by the European Commission and dedicated towards resolving some of these issues is FP7-MARINA that aims to assess, develop and optimize methodologies for life cycle analysis, exposure, hazard identification and risk assessment of NMs [14, 15]. All these efforts demonstrate the need to move towards a renovation of approaches in risk assessment [10]. In response to these needs, as well as to support the OECD Working Party on Manufactured Nanomaterials (WPMN) programme for "Testing a Representative set of Manufactured Nanomaterials", the European Commission’s Joint Research Centre (EC-JRC) established a Repository [17] that hosts different types of NMs. These NMs are representative test materials (RTM) which are defined as a material from a single batch, that is sufficiently homogeneous and stable with respect to one or more specified properties, and is implicitly assumed to be fit for its intended use in the development of test methods which target properties other than the properties for which homogeneity and stability have been demonstrated [18].

As such, the objectives of FP7-MARINA included, amongst other things, the evaluation of the robustness of current testing approaches by using a selected portfolio of *in vitro* and *in vivo* methods in the context of representative NMs. The present study is focused on the *in vitro* methodologies. The experimental approach to perform studies with multiple relevant cell types [19], or using a matrix that includes both a series of suitable cell lines and a set of standardized cytotoxicity assays [11] can represent a way to avoid false-negative outcomes and to obtain a more comprehensive view of NM biological activity. Also, depending on the mechanisms of interest, different endpoints and different sets of cell lines or co-culture models can be applied. As such, the new risk assessment strategy strives to include *in vitro* platforms which can be used for high-content data generation and computational toxicological modelling rather than relying primarily on *in vivo* studies [14, 20].

**Fig 1:**
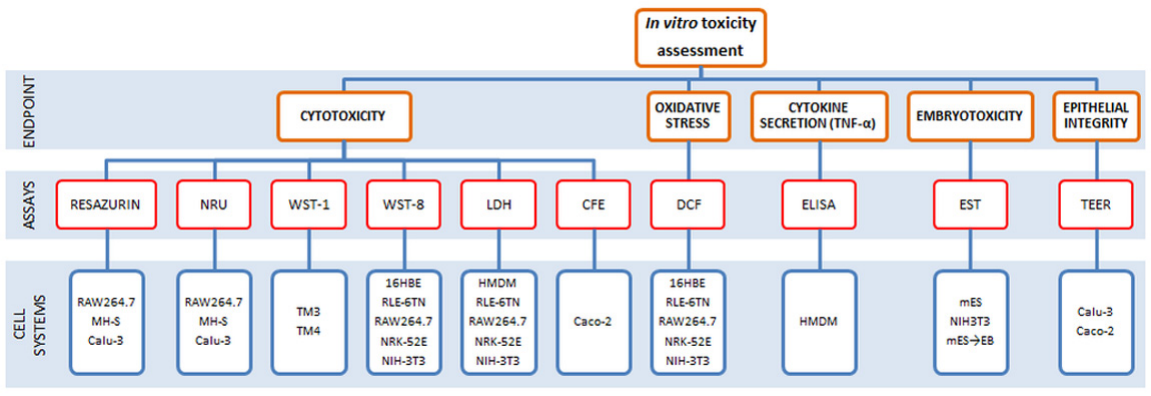
Methodology applied for the in vitro toxicity assessment. Ten different assays were used for the evaluation of cytotoxicity (resazurin, NRU, WST-1, WST-8, LDH and CFE), embryotoxicity (EST), epithelial integrity (TEER), cytokine secretion (ELISA) and oxidative stress (DCF) on twelve different cellular systems.

This study was performed on a panel of representative oxide NMs obtained from the JRC Repository: titanium dioxide (TiO_2_) (NM-103 and NM-104), zinc oxide (ZnO) (NM-110 and NM-111) and silicon dioxide (SiO_2_) (NM-200 and NM-203). The NMs were subjected to careful physico-chemical characterization. The selected assays (Fig 1) reflect different toxicity endpoints: cytotoxicity, cytokine release, oxidative stress, colony forming efficiency (CFE), epithelial barrier integrity (determined by transepithelial electrical resistance, TEER), and embryotoxicity, as evaluated using the embryonic stem cell test (EST). These endpoints were analysed using a broad range of cells representing different target systems and organs including the immune system, respiratory system, gastrointestinal system, kidneys, male reproductive system, and embryonic tissues. With this comprehensive approach, the sensitivity of the cells and of the selected assays could be evaluated and a hazard ranking of the NMs could be achieved.

## Results

### Physico-chemical characterization

The NMs (TiO_2_, ZnO and SiO_2_) selected for this study were provided by the JRC Repository. For each of these NMs two forms with different physico-chemical characteristics were selected in order to assess the influence of some specific properties on their toxicological profile. The NMs were: two representative TiO_2_ NMs with different surface chemistry due to two different coatings applied—hydrophilic (NM-103) and hydrophobic (NM-104); two SiO_2_ both uncoated, un-doped silicon dioxides produced using two different techniques: precipitation (NM-200) and pyrogenic (NM-203); the two selected ZnO NMs were uncoated (NM-110) or coated with triethoxycapryl silane (NM-111) (Table A in S1 File). Detailed descriptions of the physico-chemical properties of the NMs used in this study, as well as, the methods and SOPs used for their characterization can be found in recent JRC reports [21–23].

#### Titanium dioxide nanomaterials (TiO_2_)

NM-103 and NM-104 are homogenous within and between the examined vials and both consist of pure rutile TiO_2_. Both NMs show a small gradual thermogravimetric analysis (TGA) mass loss above 100°C indicating the presence of an organic coating. This is confirmed by Gas Chromatography—Mass Spectrometry (GC-MS) analysis which for both NMs identified the presence of different silanes. The main impurities detected by Energy Dispersive X-Ray Spectroscopy (EDS) are Al, Si, Fe and S (NM-103) and Al, Si and S (NM-104). Additionally, X-Ray Photoelectron Spectroscopy (XPS) analysis (i.e. analysis of the surface of the NM) identified the presence of Al and C for both NMs, and for NM-103 also Fe/Ca was present. Both NMs are very stable in non-acidic media and have an Iso Electrical Point (IEP) as high as 8.2 which is not typical for titanium dioxide but is due to the presence of an Al based coating on the surface. The primary particle size for both NM-103 and NM-104 is in the range 22–26 nm ± 10 nm measured by Transmission Electron Microscopy (TEM). The third dimension, height, of NM-103 and NM-104 measured by AFM was reported to be 22.3 nm and 21.8 nm, respectively (*range not given*). TEM (Transmission Electron Microscopy) analysis showed that both NMs are highly aggregated powders with aggregates in the size range of 20–500 nm and fractal like morphology. Their Brunauer, Emmet and Teller (BET) specific surface areas are around 51 m^2^/g for NM-103 and around 56 m^2^/g for NM-104, which indicate a low microporosity. The reactivity (hydrochemical pH and hydrochemical O_2_ reactivity), solubility and biodurability of TiO_2_ NMs were tested by the Sensor Disk Reader (SDR) method in three different media: 0.05% w/v BSA-water, Gamble’s solution, and Dulbecco’s modified Eagle’s medium (DMEM). No notable reactivity was observed in 0.05% w/v BSA-water for NM-103 and NM-104. In Gamble’s solution and DMEM, NM-103 acted reductive by lowering the oxygen delivery (dO_2_) value. In Gamble’s solution and DMEM, NM-104 also showed low reactivity by slightly increased dO_2_. Under *in vitro* tests conditions the experiments showed limited pH reactivity, but a burst in O_2_ concentration was observed in DMEM and Gamble’s solution for both NMs. Results from dissolution studies in the three media show that TiO_2_ is almost insoluble, whereas the Al impurities, which may originate from external coating or from the NMs themselves, are partially soluble. In addition, for NM-103 dissolved Si impurities were detected in the three media. These results suggest that the coatings of NM-103 and NM-104 may be unstable under *in vitro* test conditions.

#### Zinc oxide nanomaterials (ZnO)

NM-110 and NM-111 powders are heterogeneous NMs in both size and shape. The particles consist of both spherical and non-spherical primary particles, covering a wide size-range. NM-110 is uncoated and NM-111 is coated with triethoxycapryl silane and is hydrophobic. For NM-110, the primary ZnO crystals were observed by TEM to be polyhedral with quite variable morphology. Two main types of morphology could be distinguished: 1) particles with aspect ratio close to 1 (typically 20–250 nm size and very few particles of approx. 400 nm size) and hexagonal morphology, and 2) particles with aspect ratio 2 to 7.5 (50–350 nm) with cubic, tetragonal and orthorhombic morphologies. For NM-111 TEM analysis indicated that the primary particles are polyhedral with variable morphology and two main morphological types could be distinguished: 1) particles with aspect ratio close to 1 (~90% in the 20–200 nm range), and 2) particles with aspect ratio 2 to 8.5 (~90% in the 10–450 nm range). The mean Feret's diameter is 158 nm for NM-110 and 152 nm for NM-111.

Analyzing the NMs with Scanning Electron Microscopy (SEM) imaging, ZnO NMs have a mean primary particle diameter (mean Feret's diameter) of approximately 151 nm for NM-110 and 140 nm for NM-111 and both consist of a relatively high number of small-size particles. X-ray Diffraction (XRD) analysis indicates a hexagonal zincite structure and crystallite sizes of 41.5 and 33.8 nm for NM-110 and NM-111, respectively. TEM and SEM analysis showed that the ZnO NMs are highly agglomerated powders. The BET specific surface areas are ca. 12 m^2^/g and ca. 15 m^2^/g for NM-110 and NM-111, respectively, which indicate a low microporosity. Dissolution was measured in DI water over a period of 21 days for NM-110, resulting in concentrations of Zn^2+^ ions between 2.5 and 4 ppm. For NM-111 some data is not available (i.e. Zeta potential and solubility) due to the hydrophobic coating and difficulties in dispersing the NMs prior to analysis.

#### Silicon dioxide nanomaterials (SiO_2_)

The synthetic amorphous silicon dioxides (SAS) NM-200 and NM-203 are rather homogenous and pure consisting of 96% and 99% of SiO_2_, respectively. NM-200 contains Al (4600 ppm), Na (8800 ppm) and S (8700 ppm) impurities identified by Inductively Coupled Plasma Optical Emission Spectrometry (ICP-OES); S and Na are present as Na_2_SO_4_ which is a by-product from the synthesis. As Na_2_SO_4_ is crystalline, the presence was confirmed by XRD analysis. NM-203 contained impurities of Al (4300 ppm) and S (400 ppm) identified by EDS. The TGA analysis showed a significant mass loss below 100°C for NM-200, which may be assigned to the loss of water absorbed on the surface. NM-203 exhibits a phase transition at 324°C observed by Differential Thermal Analysis (DTA). The XPS analysis indicated presence of carbon on the surface of both NMs, which is attributed to carbon contamination on the surface of the particles.

Primary particle size of NM-200 and NM-203 measured by TEM are in the range 14 ± 7 nm and 13 ± 6 nm respectively. The third dimension, height, of the NM-200 and NM-203 particles measured by AFM is 21.9 nm and 24.2 nm respectively (*range not given*).

TEM analysis showed that NM-200 and NM-203 consist of highly porous nanostructured materials that are agglomerates and aggregates of primary particles. The pyrogenic NM-203 seems to have more complex and branched structure than the precipitated NM-200. NM-203 displays much higher angularity of spheroidal aggregates compared to NM-200.

Both NMs have SSA (specific surface area) of the same order of magnitude: 189.2 m^2^/g (NM-200) and 203.9 m^2^/g (NM-203) measured by the BET method. Small Angle X-ray Scattering (SAXS) measurements resulted in SSA of 123.3 m^2^/g (NM-200) and 167.2 m^2^/g (NM-203). Additionally, NM-200 exhibits some microporosity. The reactivity, solubility and biodurability of NM-200 and NM-203 were tested by SDR in three different media: 0.05% w/v BSA-water, Gamble’s solution, and DMEM. Under *in vitro* test conditions, hydrochemical pH reactivity tests revealed negligible to moderate effects on pH of the NM-200 and NM-203 in all three media. O_2_ reactivity tests showed some material and media-dependent effects on dO_2_ (difference between O_2_ concentration in dosed and reference vials). Increased dO_2_ values were observed for both NM-200 and NM-203 in 0.05% w/v BSA-water and Gamble’s solution, and for NM-203 also for DMEM. The variations in O_2_ concentration can be considered a strong indication of the SAS NMs to be redox-active due to direct electron transfer processes or to cause changes in the O_2_ concentration due to dissolution-related reactions. The measured 24-hour dissolution ratio revealed that both SASs and the Al impurities are partially soluble in the three media but amounts vary considerably depending on medium, as does the relative amounts of dissolved Al impurities compared with dissolved Si, suggesting that the solubility behavior of the Al impurity and SASs depend on the medium.

### Overview of toxicity assessment

**Fig 2:**
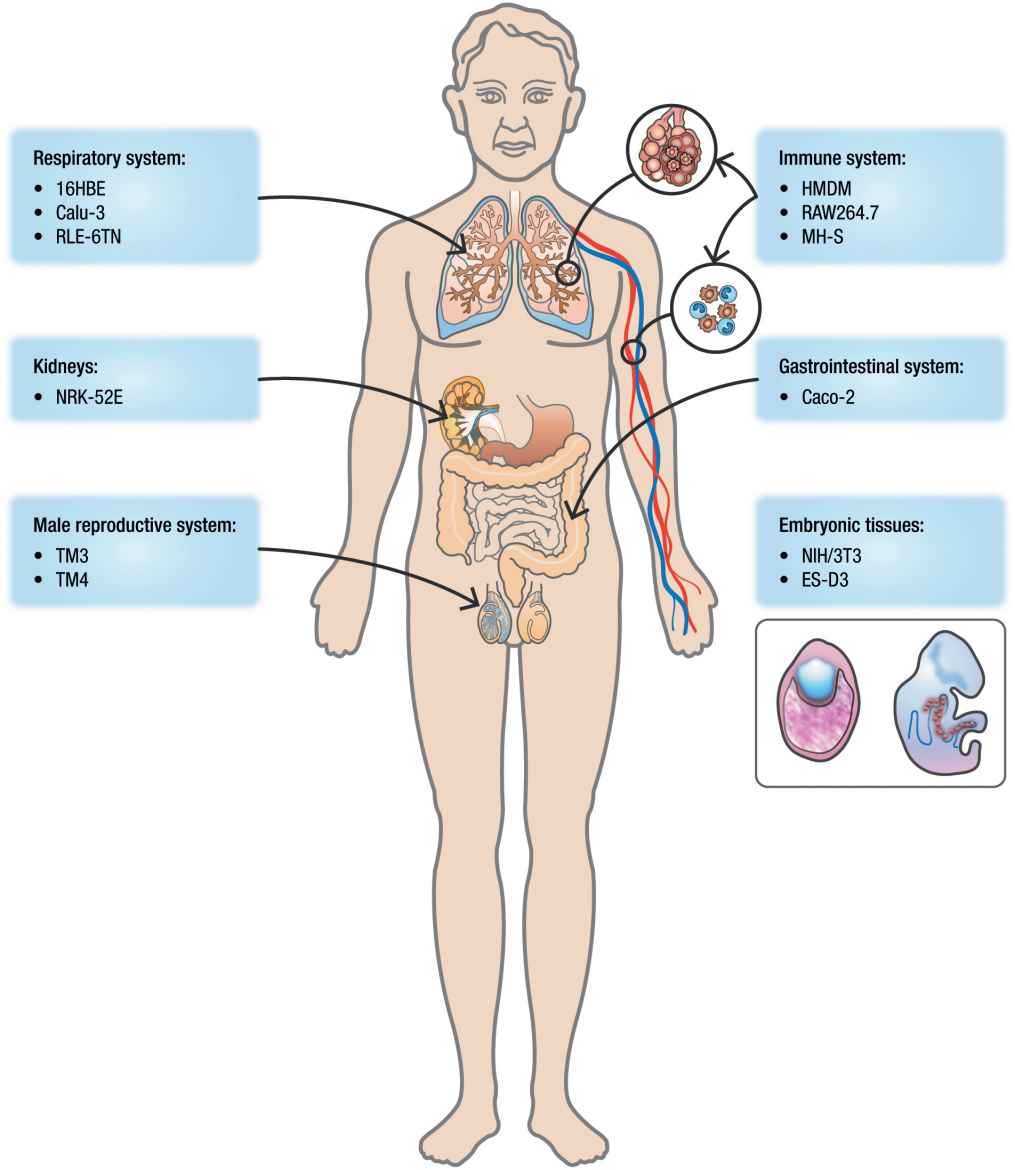
Cellular models selected for the *in vitro* toxicity study. Target organs or systems for NMs in the present study: immune system (HMDM, RAW264.7 and MH-S), respiratory system (Calu-3, 16HBE and RLE-6TN), male reproductive system (TM3 and TM4), gastrointestinal system (Caco-2), kidneys (NRK-52E) and embryo (NIH-3T3 and mES/D3).

A series of *in vitro* tests were performed in order to assess the toxic potential of the selected NMs. Ten assays reflecting the following different toxicity endpoints were used: cytotoxicity, embryotoxicity, cell-barrier integrity, inflammation and oxidative stress. These assays were performed after the exposure of different cellular systems (primary cells or cell lines) that represent important target systems for NMs (Fig 1). As such, the experimental design reflected different potential routes of exposure or target organs for NMs: the immune and respiratory systems, the gastrointestinal system, the male reproductive system, the urinary system and the embryonic tissues (Fig 2).

**Fig 3:**
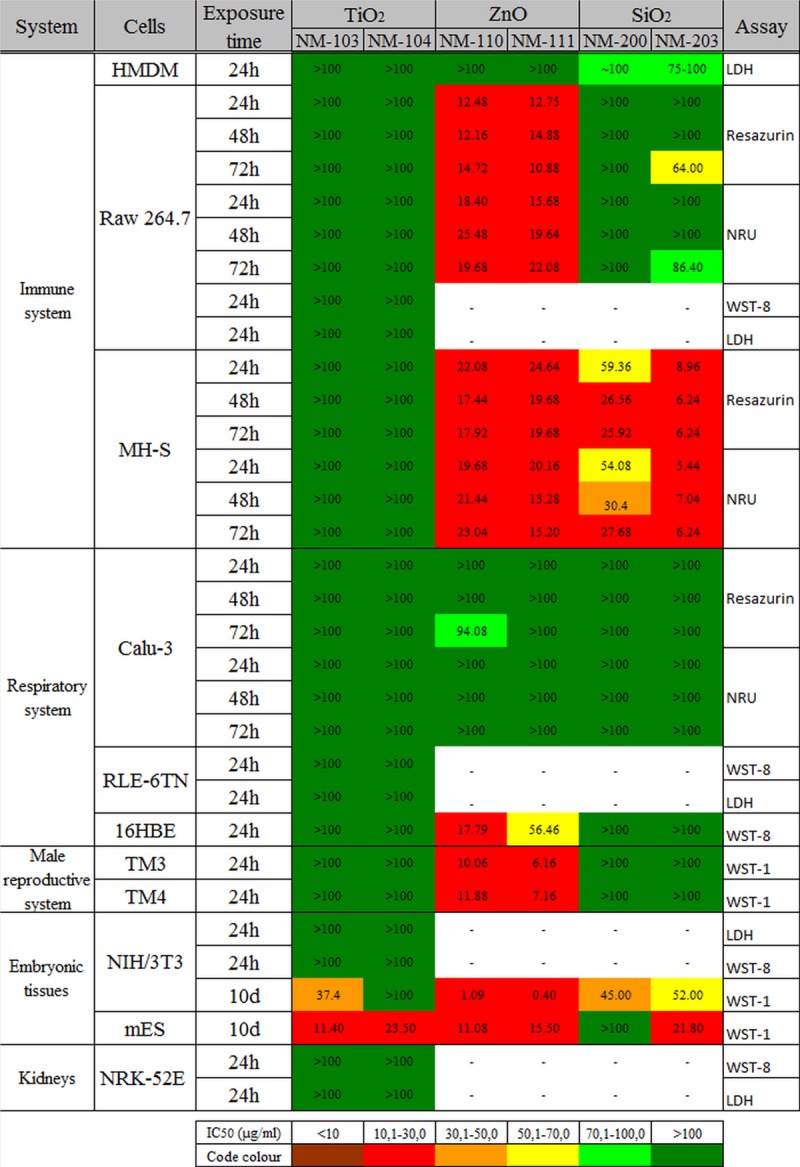
Heat map representation of IC_50_ values. The IC_**50**_ values of the six representative NMs in different cellular systems calculated at different time points (24 to 72h or 10 days). The heat map shows a higher toxicity of ZnO NMs, followed by SiO_**2**_ and TiO_**2**_. The highest sensitivity after short term exposure to NMs was noted in the case of murine alveolar macrophages (MH-S) while Calu-3 were the most resistant cells. The long term exposure (10 days) of NIH3T3 and mES cells to NMs induced also significant cytotoxic effect.

The cytotoxicity results obtained after the exposure of these cell systems to all six NMs allowed us to calculate the IC_50_ for each NM and to compare the results obtained at different time points, between different cellular systems and with different assays (Fig 3). In addition, a hazard ranking of these NMs could be established. The concentrations tested in these studies were generally between 1 and 100 μg/ml, with lower and higher limits at 0.125 and 200 μg/ml, respectively. Therefore, the concentrations to induce maximum effect (100% cell death) could not always be established, as the administration of too high doses of NMs could in some cases cause interferences with the assays. Furthermore, we have used the IC_50_ index in order to establish a hazard ranking of NMs. For this reason we have divided the results in six categories of toxicity, depending on the calculated IC_50_ value: <10, 10.1–30.0, 30.1–50.0, 50.1–70.0, 70.1–100 and >100 μg/ml. These IC_50_ values were calculated from the cell viability data by GraphPad Prism Software. The biological assays applied in this study allowed also the evaluation of different cytotoxicity mechanisms by assessing the damage of cell membrane (i.e. lactate dehydrogenase (LDH) assay), lysosomal integrity (Neutral Red Uptake) or the cellular metabolism (i.e. resazurin assay, water soluble tetrazolium—WST-1 and WST-8 assays). In addition to these colorimetric assays, we have evaluated the cytotoxicity and cytostatic effects of NMs by performing the colony forming efficiency (CFE) assay in Caco-2 cells after long term/repeated exposure. The transepithelial electrical resistance (TEER) of Calu-3 and Caco-2 cells was measured in order to evaluate epithelial barrier damages caused by exposure to NMs. The potential toxicity of all six NMs on embryonic tissues was also evaluated, by performing the Embryonic Stem Cell Test (EST). Prior to the toxicity testing, the evaluation of lipopolysaccharides (LPS) contamination using the conventional Limulus Amebocyte Lysate (LAL) chromogenic assay was performed for all NMs; the results showed that the LPS values for all samples were below the maximum admissible limit of 0.5 EU/ml [24, 25]. We also evaluated the possible interferences between the NMs and the assays reagents or the readouts and we did, indeed, observe some interference especially in the case of TiO_2_ NMs. However, this was found to not affect the outcome of the toxicity assays performed.

#### Effects on the immune system

Different types of macrophages (*i*.*e*., professional phagocytes) were selected in order to assess the toxic effects of oxide NMs towards the immune system: primary human macrophages (HMDM), murine peritoneal macrophage-like cells (RAW 264.7) and murine alveolar macrophage-like cells (MH-S).

**Fig 4:**
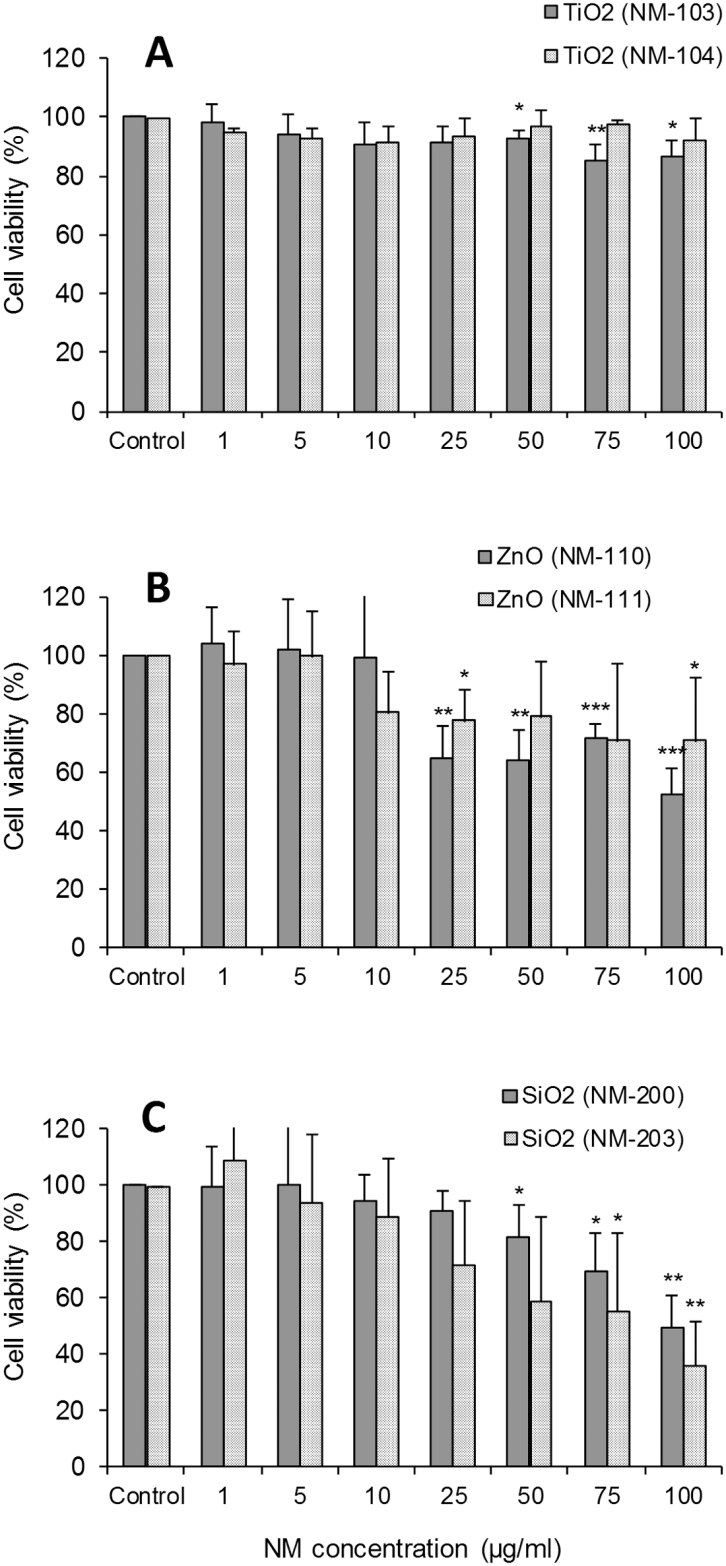
Effects on immune cells (primary human monocyte-derived macrophages). Cell viability was evaluated using LDH assay after 24h of exposure to TiO_**2**_ NM-103 and NM-104 (A), ZnO NM-110 and NM-111 (B) or SiO_**2**_ NM-200 and NM-203 (C). The results are expressed as % of cell viability (mean ± SD) versus control cells (100%) and are obtained from three to four independent experiments (donors). Statistical analysis was performed using one-way ANOVA followed by Tukey’s post-hoc test (*p< 0.05, **p < 0.01, ***p < 0.001).

A low to moderate toxicity of TiO_2_ (NM-103 and NM-104) was found in the macrophages, with all IC_50_ values >100 μg/ml. Generally, the exposure of the HMDM and RAW 264.7 cells to both types of TiO_2_ NMs did not reveal a high toxicity for these NMs, up to 72h of exposure. In the first case, in HMDM a moderate adverse effect was detected only at the highest concentrations (50 to 100 μg/ml) of NM-103. However cell viability (evaluated by LDH assay) did not decrease below 80% after 24h of exposure to any of the tested concentrations (Fig 4). Also, TNF-α secretion by HMDM was not influenced by the exposure to these NMs for 24h (Fig. A in S2 File). Similarly to HMDM, none of the two TiO_2_ NMs affected the viability of RAW 264.7 cells (evaluated by Resazurin and neutral red uptake (NRU) assays) compared with control after 24h of exposure, while after 48h and 72h a modest viability decrease was observed (Fig. B in S2 File). Additional experiments on RAW 264.7 cells using two different assays (WST-8 and LDH) confirmed the low cytotoxicity of both TiO_2_ NMs (Fig. F and Fig. G in S2 File) and effects on the ROS formation after 1h of exposure to concentrations up to 85 μg/ml (Fig. H in S2 File). The murine alveolar macrophages (MH-S cell line) were more sensitive to TiO_2_ NMs. In this case, a dose-dependent effect was observed especially after incubation with NM-104 (hydrophobic NMs) with a maximal decrease in cell viability to about 56% after 48 and 72h after exposure to 128 μg/ml (Fig. C in S2 File). These data showed a different response of the three immune cell types exposed to TiO_2_ NMs, with MH-S being the most sensitive cells. Due to this high sensitivity, in these cells we could also detect toxicity differences between the two NMs, with a slightly increased cytotoxicity for hydrophobic NM-104.

**Fig 5:**
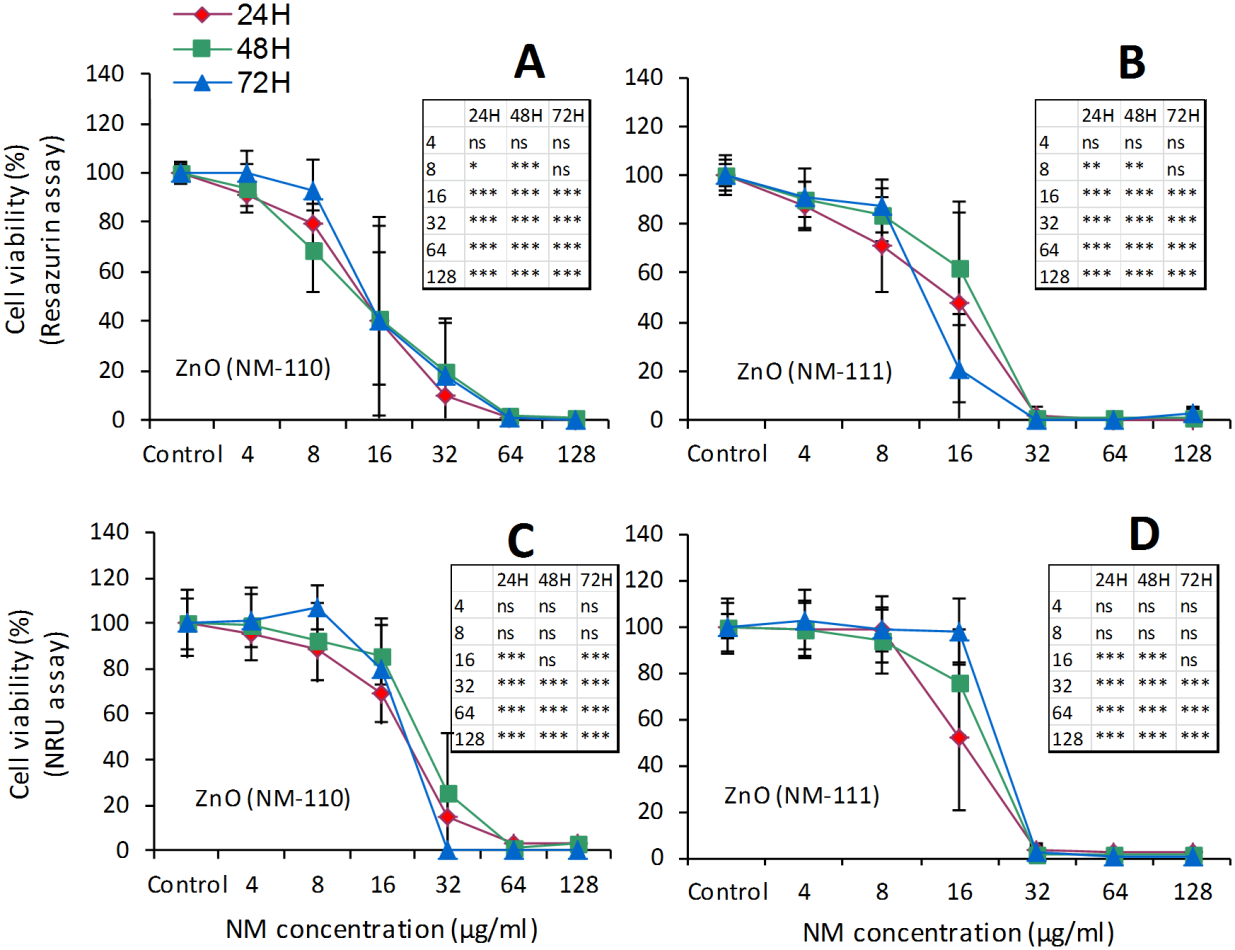
Effects on immune cells (RAW 264.7 murine macrophages). Comparative evaluation of cell viability by resazurin assay (A and B) and NRU assay (C and D) after exposure to ZnO (NM-110 and NM-111). Cells were grown for 24h in complete growth medium and then exposed for 24h, 48h and 72h to different concentrations of ZnO NMs. At the end of the incubation, cell viability was assessed using two different assays (resazurin and NRU). Data are means ± SD of 10 independent determinations in two separate experiments. Statistical analysis was performed using one-way ANOVA followed by Bonferroni post-hoc test. *p< 0.05, **p< 0.01, ***p< 0.001.

**Fig 6:**
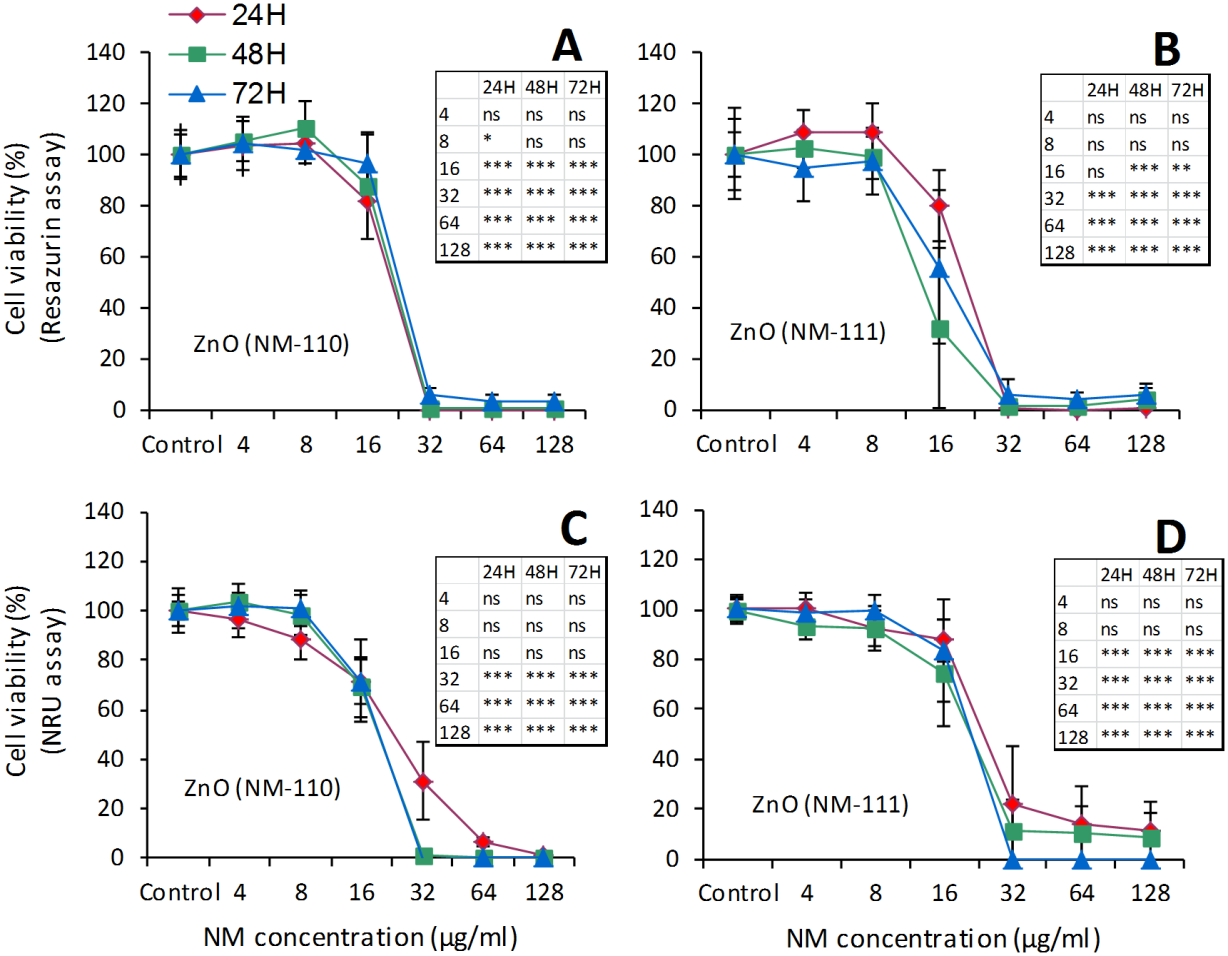
Effects on immune cells (MH-S murine alveolar macrophages). Comparative evaluation of cell viability evaluated by resazurin assay (A and B) and NRU assay (C and D) after exposure to ZnO (NM-110 and NM-111). Cells were grown for 24h in complete growth medium and then exposed for 24h, 48h and 72h to different concentrations of ZnO NMs. At the end of the incubation, cell viability was assessed using two different assays (resazurin and NRU). Data are means ± SD of 10 independent determinations in two separate experiments. Statistical analysis was performed using one-way ANOVA followed by Bonferroni post-hoc test. *p< 0.05, **p< 0.01, ***p< 0.001.

A higher toxicity of ZnO (NM-110 and NM-111) was observed in macrophages. The exposure of HMDM to ZnO NMs for 24h induced a dose-dependent decrease in cell viability (Fig 4). However, the IC_50_ values were always >100 μg/ml for both NMs. We also detected increased levels of TNF-α in the HMDM culture medium (Fig. A in S2 File) after exposure to concentrations between 5 and 50 μg/ml of NM-110 and between 1 and 25 μg/ml of NM-111. The loss of cell viability at higher doses could prevent the cytokine secretion. The RAW 264.7 cells (Fig 5) showed a marked loss of viability after exposure to ZnO NMs for 24 to 72h, which was dose- but not time-dependent. The IC_50_ values were between 10–25 μg/ml. We observed a complete loss of viability at concentrations ≥64 μg/ml for NM-110 and ≥32 μg/ml for NM-111. Similar results (IC_50_ = 15–25 μg/ml) were registered also in MH-S cells (Fig 6) with a complete loss of viability observed (resazurin assay) already after 24h of exposure to concentrations ≥32 μg/ml of both NM-110 (uncoated) and NM-111 (coated). The negative correlation between the time-points and the NM concentration observed in some cases could be explained by differences in the doses delivered to the cells in function of time or perhaps by the proliferation rate of the cells at the time-points evaluated. In other cases (i.e., for NM-111) the correlations were contradictory between the two cytotoxicity assays (NRU and resazurin). Thus, it seems a peculiar effect of the assay, highlighting the importance of applying multiple assays for testing of NMs.

The exposure of macrophages to SiO_2_ (NM-200 and NM-203) showed cell-dependent toxicity of these NMs. We observed a decrease of HMDM cell viability to 50% for NM-200 and to 36% for NM-203 at the highest concentration tested (100 μg/ml), when compared with control cells (Fig 4). TNF-α release in HMDM (Fig. A in S2 File) after exposure to SiO_2_ NMs (especially NM-200) was increased at concentrations ≥50 μg/ml. The RAW 264.7 cell viability (Fig. D in S2 File) was also affected by SiO_2_ NMs, especially in the case of NM-203. The IC_50_ at 72h was between 64–86 μg/ml for NM-203 and >100 μg/ml for NM-200. Similar exposure conditions showed a higher sensitivity for the MH-S cell line (Fig. E in S2 File) to SiO_2_ NMs with a dose-dependent decrease in viability registered after 24, 48 or 72h of exposure. The IC_50_ values were <10 μg/ml in the case of NM-203, showing a higher toxicity compared with NM-200 (IC_50_ = 25–60 μg/ml).

#### Effects on the respiratory system

Three airway epithelial cell lines (Calu-3, 16HBE and RLE-6TN) were selected for this study in order to assess the effects of oxide NMs on the respiratory system.

Exposure to TiO_2_ (NM-103 and NM-104) induced a low toxic effect to the lung epithelial cells, with IC_50_ values >100 μg/ml. In Calu-3 bronchial cells an adverse effect was observed only at the highest concentration (128 μg/ml) for both TiO_2_ NMs after 48 or 72h of exposure (Fig. I in S2 File). However, this decrease was detected by Resazurin assay but not by NRU. Additional measurements of TEER in Calu-3 epithelium after long-term exposure (up to 12 days) showed an effect on the barrier integrity in a time-dependent manner. After 12 days of exposure, the TEER was significantly decreased by 52% for NM-103 and 43% for NM-104 compared with the untreated epithelial cells. The experiments on the other two respiratory system cell lines (RLE-6TN and 16HBE) confirmed that neither TiO_2_ NM was cytotoxic after 24h of exposure. Evaluation of the ROS formation in the cell line RLE-6TN (Fig. H in S2 File), after 1h of exposure to concentrations up to 85 μg/ml, showed no significant effects compared with the negative control. On the other hand, a similar evaluation on 16HBE cells after 24h of exposure (Fig. L in S2 File) showed an increase of ROS production in these cells. However, as described above, the cellular viability of 16HBE was not influenced at this time point.

**Fig 7:**
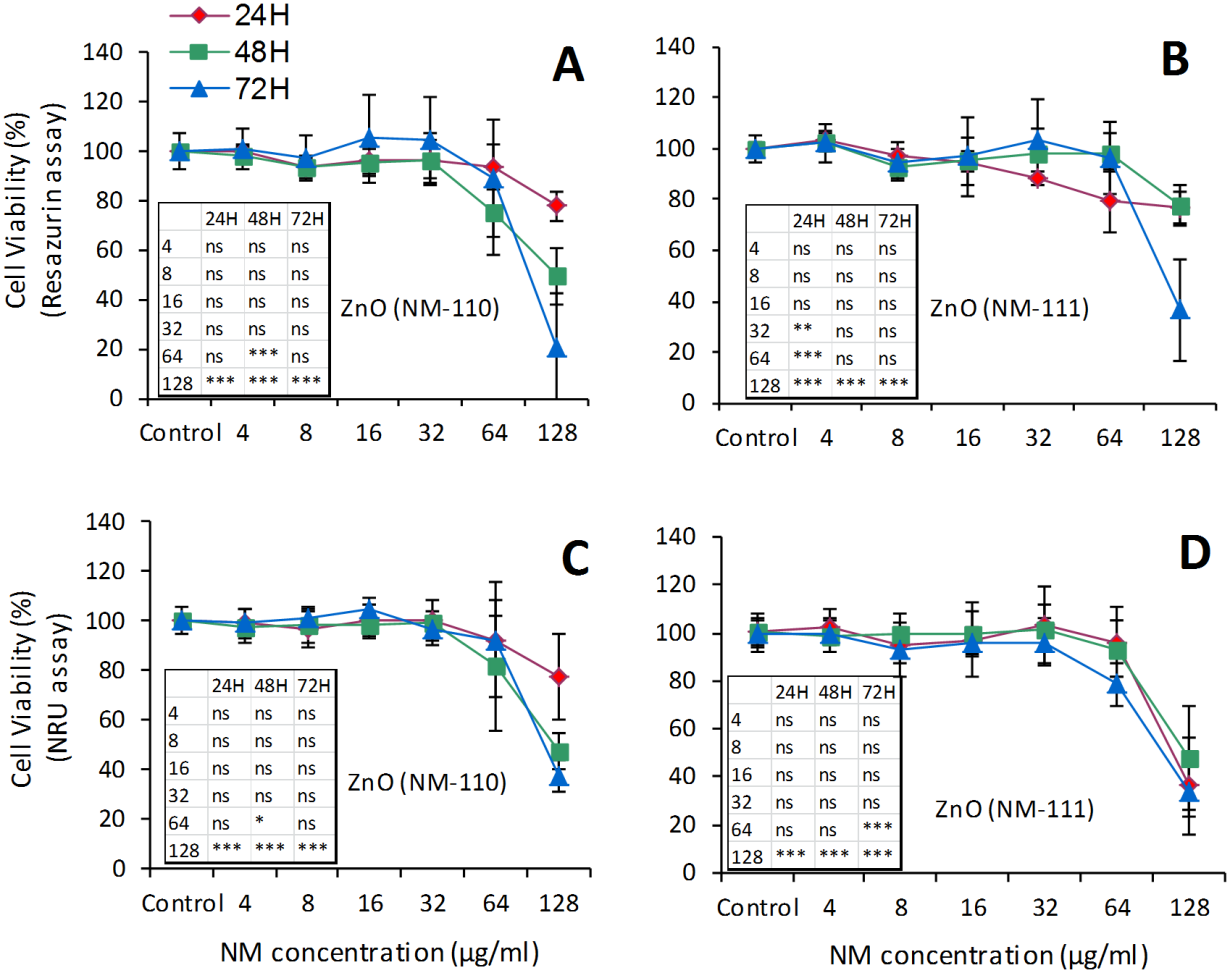
Effects on airway epithelial cells (Calu-3 bronchial cells). Cell viability was evaluated by resazurin (A and B) and NRU (C and D) assays after exposure to ZnO NM-110 and NM-111. Calu-3 cells were grown for 24h in complete growth medium and then exposed for 24h, 48h and 72h to different concentrations of ZnO NMs. At the end of the incubation, cell viability was assessed using two different assays (resazurin and NRU). Data are means ± SD of 10 independent determinations in two separate experiments. Statistical analysis was performed using one-way ANOVA followed by Bonferroni post-hoc test. *p< 0.05, **p< 0.01, ***p< 0.001.

**Fig 8:**
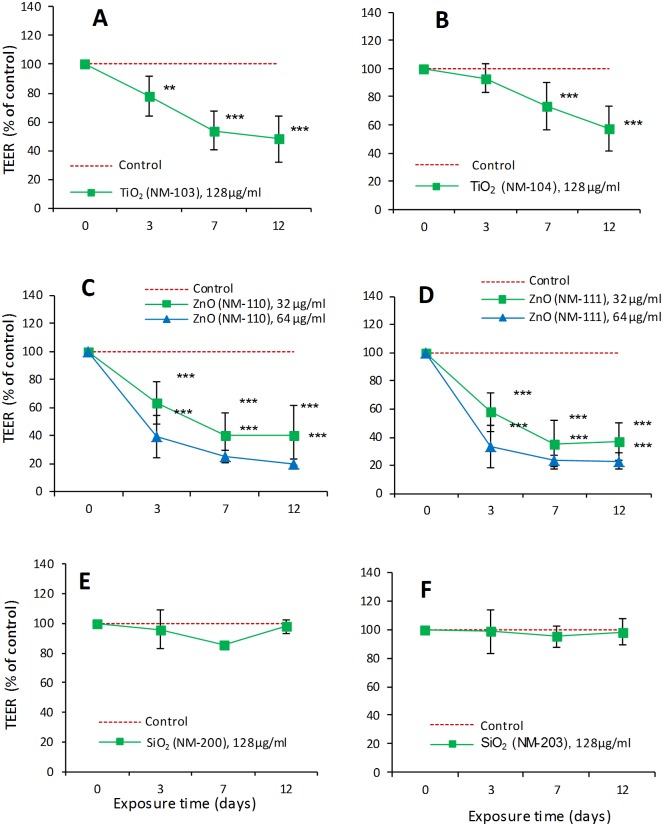
Effects on the airway epithelial cell barrier formed by Calu-3 cells. Evaluation of TEER during the exposure to TiO2 NM-103 (A) and NM-104 (B), ZnO NM-110 (C) and NM-111 (D) and SiO2 NM-200 (E) and NM-203 (F). Calu-3 epithelial cells were grown for 10 days in a double-chamber culture system to form a tight monolayer. The epithelial monolayer was then exposed at the apical side to different concentrations of NMs: 128 μg/ml (80 μg/cm²) of TiO2 and SiO2, 32 μg/ml (20 μg/cm²) and 64 μg/ml (40 μg/cm²) of ZnO. The trans-epithelial electrical resistance (TEER) was measured at the indicated times with a voltohmeter. Data are values obtained from 12 monolayers used in three separate experiments and are expressed as % of the initial value ± SD (see Methods). Statistical analysis was performed using two-way ANOVA followed by Bonferroni post-hoc test. **p< 0.01, ***p< 0.001.

The exposure to ZnO (NM-110 and NM-111) showed a cell-dependent toxicity. A difference could also be observed in terms of toxicity between the two types of ZnO NMs (uncoated NM-110 more toxic) in 16HBE but not in Calu-3 cells. Also, we observed that 16HBE cells were more sensitive to ZnO than Calu-3 cells. After 24h of exposure the IC_50_ calculated in 16HBE cells was about 17 μg/ml for NM-110 (uncoated) and about 56 μg/ml for NM-111 (coated) (Fig. K in S2 File). Similar exposure conditions also induced a significant ROS release by 16HBE cells (Fig. L in S2 File). In comparison, the IC_50_ values in Calu-3 cells were ≥100 μg/ml (Fig 7). The measurements of TEER for 12 days, following the apical exposure to 32 μg/ml (20 μg/cm²) or 64 μg/ml (40 μg/cm²) to either ZnO NM, showed a significant effect on the cell barrier at either concentration (Fig 8). After 3 days following the exposure, the TEER values were around 60% of control for both NMs. At the lower concentration (32 μg/ml) a trend toward a reduced toxicity was observed but the difference did not reach statistical significance, in comparison with NM-110.

SiO_2_ (NM-200 and NM-203) induced a low toxicity in airway epithelial cells. All calculated IC_50_ values were >100 μg/ml (Figs. J and S11 in S2 File). In addition, TEER measurements on Calu-3 epithelium exposed for 12 days to 128 μg/ml corresponding to 80 μg/cm²) confirmed the low toxicity of both SiO_2_ NMs (Fig 8). It is noted that the (modest) variation in the control values over time (eg., in Fig 8E and 8F) can be explained by the dynamic nature of this parameter; the TEER changes are plotted as the percentage of the initial value of the control cell layers. Furthermore, ROS measurements in 16HBE cells after 24h of exposure showed that both SiO_2_ NMs induced a significant ROS release (Fig. L in S2 File). However, the viability of these cells was not influenced at this time point.

#### Effects on male reproductive system

**Fig 9:**
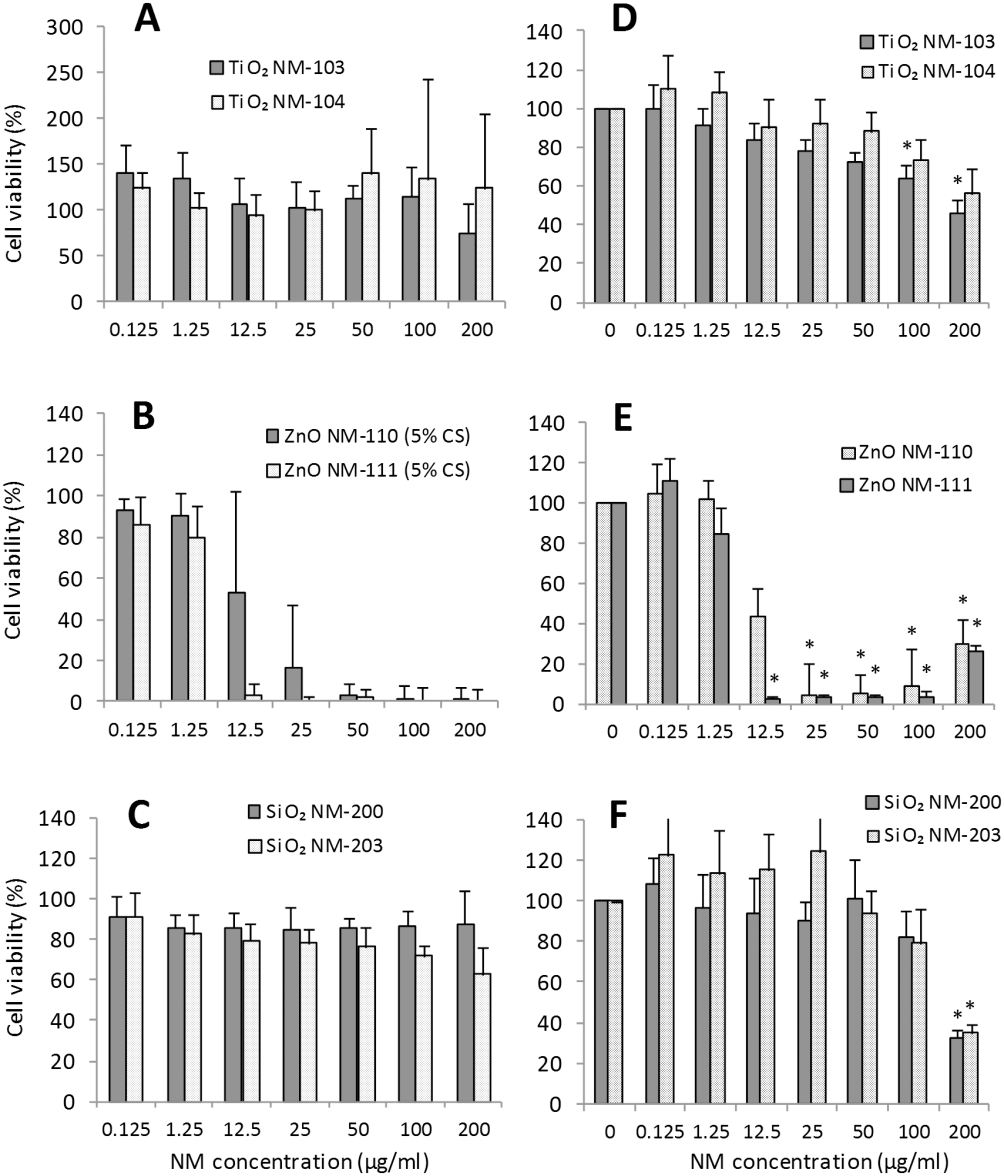
Effects on male reproductive cells (TM3 Leydig and TM4 Sertoli). The cell viability by WST-1 assay was evaluated in TM3 cells (A, B and C) and TM4 cells (D, E and F) following the exposure for 24h to TiO_**2**_ NM-103 and NM-104, ZnO NM-110 and NM-111 and SiO2 NM-200 and NM-203. The ranges of doses used were between 0.125 μg/ml (0.037 μg/cm^2^) and 200 μg/ml (60.24 μg/cm^2^). WST-1 assay measurements were taken (120 min post addition). All results were corrected for WST-1, as the control showed presence of interference in absorbance reading as a result of WST. The statistical analysis of the data (n = 6) was performed by one way ANOVA, Dunnett's multiple comparison test and Tukey’s post-hoc test (p<0.001*).

Two testicular cell lines (mouse TM3 Leydig and TM4 Sertoli) were used to evaluate the cytotoxic effects of oxide NMs on the male reproductive system (Fig 9).

The exposure to TiO_2_ (NM-103 and NM-104) induced a moderate toxicity to the testicular cells. The IC_50_ values calculated after 24h of exposure were >100 μg/ml. Cytotoxicity evaluation on Leydig TM3 cells showed a change in cell viability for NM-103 (73% when compared with the control) only after exposure to a high concentration (200 μg/ml), while there was little alteration in viability across the range of doses tested for NM-104. The Sertoli TM4 cells were not affected by NM-103 and NM-104 at doses <50 μg/ml after 24h of exposure. However, at 100 and 200 μg/ml the effects were statistically significant in the case of NM-103, with a decrease of viability to 63% and 45%, respectively. Equivalent doses of NM-104 induced also a decrease of cell viability to 73% and 55%, respectively.

A high toxicity of ZnO (NM-110 and NM-111) was observed in testicular cells, with IC_50_ values around 10 μg/ml. For NM-110, the results show a high degree of variability among replicates of the assay at 12.5 μg/ml. We identified this as the dose around which a significant drop in viability was observed, but the variation in response was so wide that toxicity was not statistically significant until 25 μg/ml. For NM-111, we also observed a significant drop of cell viability at concentrations ≥12.5 μg/ml. In the TM4 cell line a complete inhibition of cell viability was observed starting from a concentration of 25 μg/ml for NM-110 (uncoated) and of 12.5 μg/ml for NM-111 (coated).

SiO_2_ (NM-200 and NM-203) caused low toxicity in testicular cells. The TM3 cells were not affected by the exposure to NM-200, while NM-203 induced a decrease in cell viability at doses ≥100 μg/ml. Similarly, in TM4 cells an effect on cell viability was observed only after exposure to concentrations ≥100 μg/ml. However, the IC_50_ values being ≥100 μg/ml in all cases.

#### Embryotoxicity

**Fig 10:**
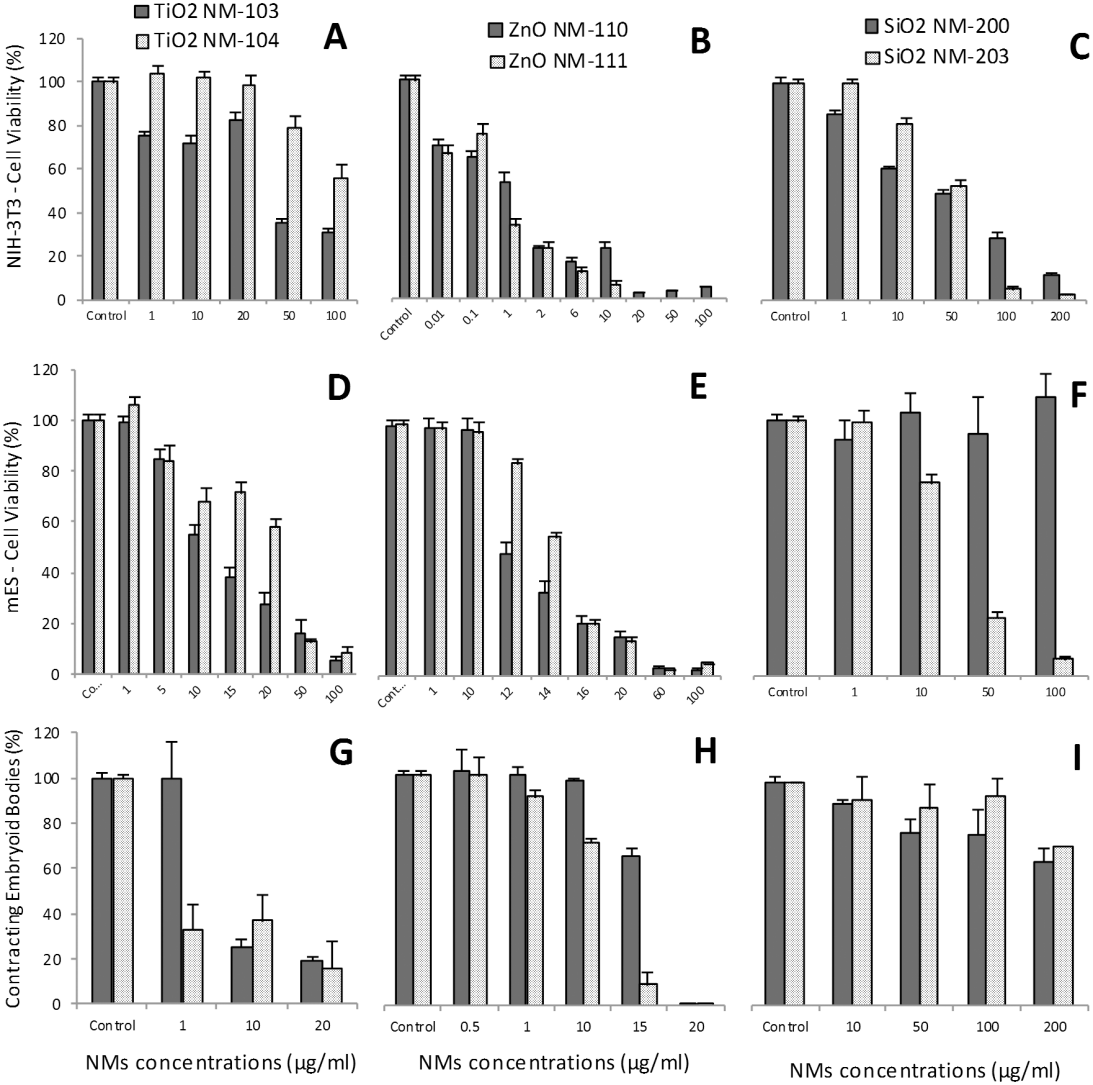
Embryotoxicity evaluation by Embryonic Stem Cell Test (EST). The following parameters were used for EST: viability of NIH-3T3 (A, B, C) and mES cells (D, E, F), respectively the mES differentiation into contracting EBs (G, H, I). The cell viability was evaluated by WST-1 assay on day 10 after exposure and the cell differentiation was assessed by direct visualization of beating areas under a light microscope after exposure to different concentrations of TiO_**2**_ (NM-103 and NM-104), ZnO (NM-110 and NM-111) or SiO_**2**_ (NM-200 and NM-203). The values represent the means ± SE of at least four independent experiments.

Two stable cell lines were used in the Embryonic Stem Cell Test (EST): NIH3T3 fibroblasts and mouse embryonic stem (mES) cells, representing differentiated and undifferentiated tissue, respectively. Three endpoints are obtained from dose/response curves after 10 days of culture (Fig 10 and Table 1): *(i)* 50% inhibition of differentiation of mES cells into contracting myocardial cells (ID_50_), as assessed by morphological analysis of beating cell areas (contracting embryoid bodies, EBs), *(ii)* 50% inhibition of proliferation of mES (IC_50_ES) and *(iii)* of NIH3T3 cells (IC_50_3T3). Furthermore, a mathematical algorithm that integrates these three values is used to obtain an evaluation of predicted embryotoxicity [26].

**Table 1:** Values of ID_50_ and IC_50_ obtained after exposure of mES and NIH3T3 cells to NMs.

	TiO_2_		ZnO		SiO_2_	
	NM-103	NM-104	NM-110	NM-111	NM-200	NM-203
**IC**_**50**_ **3T3 (μg/ml)**	37.4	>100	1.09	0.4	45	52
**IC**_**50**_ **ES (μg/ml)**	11.4	23.5	11.08	15.5	>100	21.8
**ID**_**50**_ **(μg/ml)**	4.6	<1	15.9	11.5	>100	>100
**Embryotoxicity classification**	Weak-embryotoxic	Weak-embryotoxic	Non-embryotoxic	Non-embryotoxic	Non-embryotoxic	Non-embryotoxic

For TiO_2_ NM-103, the IC_50_3T3 and the IC_50_ES were 37.4 μg/ml and 11.4 μg/ml, respectively. The ID_50_ was 4.6 μg/ml. The corresponding values for TiO_2_ NM-104 were: >100 μg/ml, 23.5 μg/ml, and < 1 μg/ml.

The IC_50_3T3 value after exposure to ZnO NM-110 was 1.09 μg/ml, while the IC_50_ES was 11.08 μg/ml for mES cells. The ID_50_ was 15.9 μg/ml. The corresponding values for ZnO NM-111 were: 0.4 μg/ml, 15.5 μg/ml and 11.5 μg/ml.

The IC_50_3T3 after exposure to SiO_2_ NM-200 was 45 μg/ml while the IC_50_ES and ID_50_ were >100 μg/ml. The corresponding values for SiO_2_ NM-203 were as follow: 52 μg/ml, 21.8 μg/ml, and >100 μg/ml.

Based on the EST mathematical predictive model, the TiO_2_ NMs were classified as “*weak-embryotoxic*” while ZnO and SiO_2_ NMs as “*non-embryotoxic*”.

Additionally to EST, a short term exposure (up to 24h) to TiO_2_ NMs was performed in NIH/3T3 cells. Two parameters, cell viability at 24h and ROS production at 1h, were evaluated. At all the concentrations tested, up to 170 μg/ml, no cytotoxicity was recorded (Figs. F and G in S2 File); regarding ROS production, no significant effect was observed for concentrations up to 85 μg/ml (Fig. H in S2 File).

#### Effects on other organs or systems (intestinal and kidney cell models)

**Fig 11:**
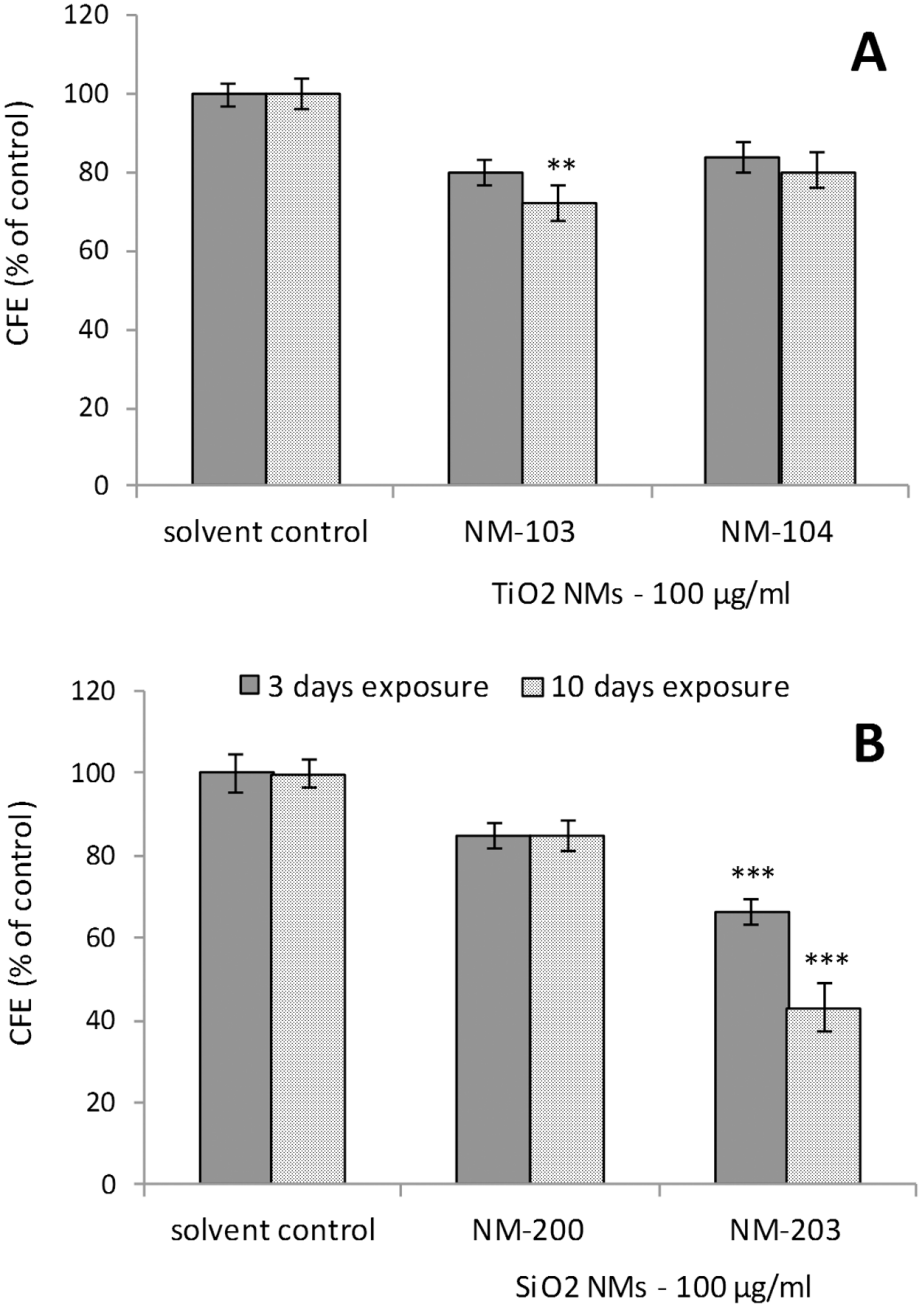
Effects on the gastrointestinal tract (Caco-2 intestinal cells). The cytotoxic and cytostatic effects were evaluated by Colony Forming Efficiency Assay (CFE) in Caco-2 cells exposed to 100 μg/ml of TiO_**2**_ (NM-103 and NM-104) (A) and SiO_**2**_ (NM-200 and NM-203) (B) for 3 (short exposure) and 10 days (long term, repeated-dose exposure). The results are expressed as CFE (% of solvent control) = ([average No. of treatment colonies/average No. of solvent control colonies] × 100). The solvent control (0.05% BSA) did not induce cytotoxicity. Data are reported as means ± SEM (standard error mean = standard deviation/√number of replicates). One-way analysis of variance (ANOVA) with post hoc test (Dunnett's Multiple Comparison Test) for comparing groups of data versus one control group was used (** p<0.01; *** p<0.001).

Caco-2 cell line (intestinal epithelial cells) was used in order to evaluate the effects of repeated exposure to oxide NMs on the cell barrier and also to perform a toxicity screening following long-term and repeated exposure to the same NMs. However, neither TiO_2_ (NM-103 and NM-104) nor SiO_2_ (NM-200 and NM-203) induced any changes in Caco-2 epithelium TEER values during the 21 days of monitoring (7 repeated exposures at a concentration of 100 μg/ml) (Fig. M in S2 File). CFE assay (Fig 11) showed that short-term exposure (3 days) to both TiO_2_ NM-103 and NM-104 did not induce statistically significant changes in the colony forming efficiency and average colony area with respect to control. Therefore, we concluded that no cytotoxic or cytostatic effects were induced by these NMs in Caco-2 cells. When the cells were exposed to repeated doses of TiO_2_ NMs over 10 days, statistically significant cytotoxic and cytostatic effects were detected in respect to control only after exposure to NM-103 (cytotoxic: CFE = 72% ± 5; cytostatic: Average area = 81% ± 3). In case of short term exposure to SiO_2_ NMs, statistically significant cytotoxic and cytostatic effects were registered only for NM-203, with significant values of cytotoxicity (CFE = 66% ± 4) and cytostaticity (average area = 72% ± 3). These statistically significant effects were even more evident after repeated exposures to NM-203 over 10 days for both, cytotoxicity (CFE = 43% ± 4) and cytostaticity (Average area = 41% ± 1).

NM effects on kidney cells were evaluated by using the NRK-52E cell line (normal rat kidney) exposed to TiO_2_ (NM-103 and NM-104) to concentrations up to 170 μg/ml. None of the two TiO_2_ NMs significantly reduced viability of the kidney cells (Figs. F and G in S2 File). Similarly, no ROS (Fig. H in S2 File) formation was detected after 1h exposure to either NM-103 or NM-104.

## Discussion

### Testing of representative nanomaterials

One of the key features of the present study, which was undertaken in the frame of the EU-funded FP7-MARINA project, was to apply a panel of representative NMs [TiO_2_ (NM-103 and NM-104), ZnO (NM-110 and NM-111) and SiO_2_ (NM-200 and NM-203)] from the JRC Repository across a range of test systems. In addition, these materials are also used in several major international research programs, such as the OECD Testing Programme [27], NANOGENOTOX Joint Action [28] and a multiplicity of research projects, including FP7-NANOREG [29]. By definition, a representative test material (RTM) is not a reference material for the test for which it is intended to be used, because homogeneity and stability are not demonstrated for the corresponding measurand [18]. However, an RTM is more valuable than an ordinary test material, since it has been checked for homogeneity and stability in terms of one or more specified properties. RTMs are extremely useful tools in intra- or inter-laboratory development of methods for which reference materials are not available. In this context, RTMs might be useful tools for testing of variability across laboratories in regard to the physico-chemical properties of NMs [30]. As already demonstrated, the biological impact of NMs results from the combined effects of a variety of physico-chemical properties including chemical composition, size, surface coating and surface chemistry [11]; therefore, the use of RTMs is important in order to reliably address the scientific questions on toxicity effects of NMs in a comparable and reproducible way. Such RTM allow enhanced comparison of test results and pave the way for appropriate test method optimization, harmonization and validation and may eventually serve as performance standards for testing. For nanosafety research purposes, availability of NMs from a single batch is desirable to enhance the comparability of results among different laboratories and research projects. Such availability would overcome questions related to whether a NM tested in one project is the same or just similar to a NM tested in other project(s) and how the results are compared between different studies [18].

### Methodology assessment

The selection of the most suitable *in vitro* tests methods to be used for NM toxicity testing and of the most appropriate approach to exploit the results obtained for hazard identification still remains a challenge for risk assessors, scientists, industry and regulators. Although there are many studies addressing the toxicity of NMs, there is no standardized testing procedure that can be generally applied to all types of NMs. However, the final goal of such studies is to provide reliable information on the risk that the NMs may give rise to within their life-cycle and, finally, to assure the safety of all production workers and end-users. As previously suggested, an *in vitro* testing strategy based on a matrix that should include a series of suitable cell lines and a set of standardized cytotoxicity assays might be a useful approach in order to obtain a more comprehensive view of NM biological activity [11, 19]. Such a testing approach on a panel of RTMs, as in our study, can further enhance the reliability and the utility of the data generated. In our study, the *in vitro* testing approach covered a panel of different important endpoints for toxicity assessment (cytotoxicity, embryotoxicity, epithelial cell-barrier integrity, cytokine release and oxidative stress). These endpoints were evaluated by using ten different *in vitro* assays (Fig 1). Most of the methods applied are designed to evaluate the (cyto)toxic effects of chemicals and the applicability for NMs was not yet completely assessed [31]. These assays are usually performed using colorimetric or fluorescent dyes as markers to determine cell viability assessing plasma membrane integrity (i.e. LDH assay), lysosome integrity (NRU assay) or cellular metabolism (i.e. Resazurin and WST-8 assays). While these assays have been found to provide accurate viability data for classic small molecule cytotoxicity studies, their reliability for evaluating the toxicity of NMs still has not been demonstrated yet [32, 33]. However, *in vitro* assays seem to be able to detect the toxic or inflammatory potential of NMs that act via soluble ions (i.e. ZnO, CuO) [34]. Another methodology that we have applied consisted in the evaluation of toxic effects of NMs on embryonic tissues. The Embryonic Stem Cell Test (EST) is a validated test for predicting embryotoxicity of chemical compounds [35] and its possible extension to NMs has been recently reported [26, 36].

In regard to the sensitivity of the assays, in our study we observed that the NRU assay appeared to be less sensitive compared with resazurin in detecting decrease in cell viability upon exposure to all six NMs, an observation confirmed by the comparative IC_50_ values obtained with the two methods. This comparison was performed using three different types of cells (RAW 264.7, MH-S and Calu-3).

In addition to the colorimetric assays, two functional and non-colorimetric methods were performed. TEER (Transepithelial Electrical Resistance), for the estimation of epithelial cell-barrier damage, and CFE (Colony Forming Efficiency), which is designed to assess the cytotoxic and cytostatic potential of tested materials.

Prior to the toxicity assessment, experiments were performed in order to evaluate the possible interference of the selected NMs with the *in vitro* assays (see S3 File). As pointed out previously, the NM-assay interferences are not always reported [37]. Therefore, we have studied the possible interferences of the NMs with the assays. Thus, we noted an increase in the OD readings, especially for the NRU, LDH and WST-8 assays due to the presence of NMs (*e*.*g*. titanium oxide), in agreement with previously published data on similar NMs [33, 38]. Indeed, Kroll et al. [38] have suggested that each in vitro test system has to be evaluated for each NM to accurately assess for possible interferences. However, while a dose-dependent interference was observed for some nanomaterials with some of the assays (*i*.*e*. TiO_2_ in LDH assay), this effect was minor (1–3%) (see Figs. O-U in S3 File). Therefore, the observed interferences did not significantly affect the outcome of the toxicity testing.

### Toxicity assessment of oxide nanomaterials

The strength of the present study consists in the use of the same (six) representative oxide NMs throughout all the testing procedures. The toxicity was evaluated using various cell systems and different assays, focused mainly on the cytotoxic effects. Comparative observations on cell sensitivity, assay reliability, influence of the physico-chemical properties on the toxicity and possible NM-assay interferences were also reported. The potential of nanoparticles triggering cellular pathways is largely reliant on their surface chemistry however, this is also dependent on the exposure conditions and the dose accumulated in cells. The presence of physiologically relevant protein concentrations reduces the nanoparticle dose and thus modulates nanoparticle induced cytoxicity [39, 40]. The role of adsorbed biomolecules derived from the environment significantly modifies the nanoparticle surface in such a manner that the bionanointeractions are radically different from those suggested by the bare material itself. Therefore, the nanomaterials investigated herein are studied *in situ*.

Titanium dioxide (TiO_2_) NMs are some of the most abundantly produced NMs and are found in diverse every-day and nanotechnology-enabled products and applications. Their wide use in applications ranging from cosmetics and sunscreens to heterogeneous catalysts results in an increased likelihood of either intentional or unintentional exposure [41]. Within the efforts to elucidate possible hazardous effects of titanium NMs, recent studies [42] using mouse macrophages showed a size dependent cytotoxicity and increased oxidative stress levels after exposure to anatase TiO_2_ (anatase) NMs. However, to date, there is no unanimous conclusion about the impact of particle size on the toxicity of TiO_2_ NMs. In our study, TiO_2_ (rutile) NMs showed minor effects to most of the cells studied, up to 72h of exposure. These results are in agreement with previous studies [43] that are suggesting a lower bioreactivity for rutile TiO_2_ compared with the anatase form, but also a higher sensitivity of MH-S cells (alveolar macrophages) compared with other cell types [42]. In this context it is relevant to mention that both TiO_2_ NMs were coated with Al. On the other hand, the dissolution experiments showed that both NMs are insoluble, while the Al coatings may be unstable under *in vitro* test conditions. Our results could not establish a consistent difference between the hazardous properties of the NM-103 (hydrophilic) and NM-104 (hydrophobic) in all the cell models adopted; a difference could be observed only in the case of alveolar macrophages (MH-S), with a slight increase of cytotoxicity for hydrophobic NM-104. This observation may emphasize the need to use a panel of different cell lines (with different sensitivity) for NMs toxicity assessment. The low toxicity of NM-103 and NM-104 after short-term exposure was confirmed by the test results obtained on all the other cell types used in this study. Notably, a different situation was observed after longer exposures (≥10 days). Both NM-103 and NM-104 displayed some toxicity on the mouse embryonic stem cell line D3 (mES) and on mouse NIH/3T3 fibroblasts. A difference could be also observed between the two NMs, with a higher toxicity for NM-103 (hydrophilic) to both mES and NIH/3T3. The aim of this 10-day *in vitro* study was to evaluate the embryotoxic potential of TiO_2_ and, by applying the EST algorithm, it was concluded that both TiO_2_ NMs can be classified as “*weak-embryotoxic*”. Additionally, a long term exposure (10 days) or repeated exposure (7 exposures within 21 days) of Caco-2 intestinal cells to both TiO_2_ NMs induced minor toxic effects, at least in the *in vitro* assays used in this study. Moreover, marked changes in TEER were observed in Calu-3 cell monolayers exposed to either NM-103 or NM-104.

Zinc oxide (ZnO) NMs are already produced in high tonnage and their intentional use in commercial applications, such as antibacterial coatings or UV absorbers in sunscreens and textiles, requires immediate knowledge concerning their toxic potential [44]. Recently, ZnO NMs have been suggested as potential anticancer agents due to their preferential toxicity towards rapidly dividing cancerous cells [45]. Several toxicological studies have shown that ZnO NMs significantly reduced cell viability not only in cancer cell lines of the lung, kidney, skin, immune system or the gut but also in primary cells including immune cells, neural stem cells or fibroblasts [44, 46]. Other adverse effects that have been ascribed to ZnO NMs include inflammatory reactions, altered cell cycle and DNA damage [44, 47]. However, when the aim is to identify the key physico-chemical properties that might be responsible for the high toxicity of ZnO NMs, no clear characteristic that governs cytotoxicity has been described so far. As already described [44], coating of ZnO may prevent the release of Zn^2+^ ions, which presumably are responsible for the toxicity of the ZnO NMs. As such, we have tested two types of ZnO, NM-110 that is uncoated and NM-111, that is coated with hydrophobic triethoxycapryl silane. Moreover, we have measured the dissolution in DI water over a period of 21 days. For NM-110 the concentrations of Zn^2+^ ions were between 2.5 and 4 ppm. For NM-111 we could not establish these values due to the hydrophobic coating and difficulties in dispersing the NM prior to analysis. However, both NMs were highly toxic to all cellular system tested. A difference regarding their cytotoxicity (NM-110 > NM-111) could be observed in the immune cells (HMDM) and respiratory epithelial cells (16HBE), indicating that the coating of ZnO NMs (with triethoxycapryl silane) may influence the toxic effects. The findings in HMDM (primary cells) were not confirmed in the two macrophage cell lines, i.e. RAW 264.7 and MH-S, indicating that primary cells and cell lines differ in their response to NMs [46]. The exposure of the embryonic stem cells (mES) for 10 days to NM-110 and NM-111 showed a high toxicity of both types of ZnO NMs, with respect to cell viability; however, the effect of both forms of NMs on the differentiation of embryonic cells to contracting cardiomyocytes was evident only at the higher concentrations tested. Following the application of the EST algorithm, the ZnO NMs were classified as “*non-embryotoxic*”.

Silicon dioxide (SiO_2_) NMs are used in different industrial or consumer products as additives to *e*.*g*. cosmetics, drugs, printer toners, paper, tyres, varnishes or food but also for biomedical and biotechnological applications. Most of the *in vitro* studies to date report size- and dose-dependent cytotoxicity, increased ROS levels, production of pro-inflammatory mediators, and cellular uptake of these NMs [48]. The complexity of protein-SiO_2_ NMs interactions also appears to be affected by size, while the (eco)toxicological effect(s) of other physico-chemical properties of SiO_2_ NMs, such as porosity, chemical purity, surface chemistry and solubility, are less well studied. Previous studies [49] suggested a prominent role for lipid peroxidation at the plasma membrane and/or at intracellular sites for SiO_2_ NM-induced cytotoxicity. The adverse effects of the SiO_2_ NMs were reversed in the presence of serum, possibly due to the masking of reactive surface groups.

The SiO_2_ NMs used in our study were produced by two different manufacturing techniques, precipitated (NM-200) and pyrogenic method (NM-203). Even though both NMs have similar physico-chemical characteristics, NM-203 displays a much higher angularity compared to NM-200. A difference could be observed also in regards to the hazardous properties, as NM-203 was generally more toxic than NM-200. This difference could be observed in all the immune cells (MH-S cells were the most sensitive) after 24-72h of exposure but also after long-term exposure (10 days) of embryonic cells NIH/3T3. Higher toxicity of NM-203 was observed also in mES, with respect to cell viability; however, similarly to ZnO NMs, differentiation of embryonic cells to contracting cardiomyocytes was only slightly impaired. Applying the EST algorithm, both NMs were classified as “*non-embryotoxic*”.

Similar exposure for 10 days of Caco-2 cells showed statistically higher cytotoxic and cytostatic effects of NM-203 when compared with NM-200. On the other hand, neither NM-200 nor NM-203 induced any observable toxic effect on cells of the male reproductive system (TM3 Leydig and TM4 Sertoli cells) or of the lung (16HBE and Calu-3 cells), up to 100 μg/ml.

### Towards an intelligent testing strategy for nanomaterials

In order to apply *in vitro* test systems in a risk assessment strategy for NMs, the test systems should meet certain technical preconditions, *e*.*g*. they should be without assay-specific interferences [33]. This has been investigated here for a number of *in vitro* tests. Furthermore, it is of importance to verify whether a negative test outcome indicates a non-toxic or very low toxic potential or whether the absence of ‘toxicity’ could be explained, for instance, by the fact that the NM did not reach the cell target to exert a toxic effect. For example, a NM might form aggregates/agglomerates through interaction with the test medium which could preclude its interaction with subcellular targets. Other issues that need to be addressed are the physico-chemical characterization of the nanomaterials in the test system and the issue of the cellular dose. Indeed, as pointed out by Cohen et al. [50] the dose that is actually delivered to cells in an *in vitro* setting may not be accurately reflected by the administered dose of particles in suspension. Furthermore, it should be possible to extrapolate the behavior of the NM in a test system and the resulting hazard estimation to the human situation. The corroboration of results obtained using *in vitro* tests in animal models should be considered (but this has not been a part of the present study). The main question relevant for the development of an ITS and risk assessment strategy for NMs is to establish if an *in vitro* assay or a battery of *in vitro* assays provides sufficient information on the relevance of a specific endpoint or concern or whether potential effects, *e*.*g*. effects caused by a mode-of-action not covered by the test system or set of test systems have been overlooked. Another highly relevant issue for an ITS or risk assessment strategy is the possibility of ranking materials, either preparations of the same material with different physico-chemical properties due, for example, to changes during the life-cycle, or a collection of NMs that may be relevant for grouping [6, 7].

## Conclusions

The results obtained allow a hazard ranking of the six oxide NMs tested as follows: ZnO NM-110 > ZnO NM-111 > SiO_2_ NM-203 > SiO_2_ NM-200 > TiO_2_ NM-104 > TiO_2_ NM-103. This ranking is based on the results obtained after short term exposures (24-72h) using a broad range of test systems. The physico-chemical properties played a role in the expression of NM toxicity. For example, the presence of coating on ZnO NM-111 was correlated in some of the studies with a lower toxicity when compared with the uncoated ZnO NM-110. Also, the higher angularity of SiO_2_ NM-203 increased its toxicity when compared with SiO_2_ NM-200.

A cell-specific response to NMs was observed in the present study. Hence, upon short term exposure (24-72h), immune cells showed a higher sensitivity to NMs, followed by testicular cells and airway epithelial cells. Overall, the most sensitive cells studied were the murine alveolar macrophages (MH-S). Notably, however, the assessment of embryotoxicity using the EST classified the two TiO_2_ NMs as ‘*weak embryotoxic*’, and both ZnO and SiO_2_ NMs as ‘*non embryotoxic*’. Further studies in order to elucidate the applicability of EST for embryotoxicity assessment of NMs are therefore needed. In general, data obtained within our studies showed consistent results between different methods applied. For the assays that focused on the same mechanism (*e*.*g*. mitochondrial dehydrogenase activity, as a marker of cell viability), the results showed good reproducibility. Moreover, also when assays focused on different endpoints were applied, the results were, overall, consistent (Fig 3). The interferences with the assays that were observed for some of the NMs (especially for TiO_2_) were considered non-significant.

In summary, based on the present, multi-laboratory study, we suggest that a test battery designed to be included in an intelligent testing strategy (ITS) suitable for risk assessment of NMs should include:

1. Cellular systems with biological relevance for targets in the human body (more than one target), including more sensitive cell models (such as macrophages) that can detect possible adverse effects at low doses that are potentially more relevant for human exposure;
2. Reliable test assays, with no NMs interferences, designed to capture relevant mechanisms of toxicity. In addition, more complex test methods (*i*.*e*. embryonic stem cell test) and label-free test methods (*e*.*g*. the TEER method for barrier integrity) should also be considered;
3. Thorough physico-chemical characterization of NMs in order to interpret the toxicity data; indeed, further studies of structure-activity relationships of NMs are needed, and the present, comprehensive study provides a rich source of data for such QSAR-modeling [51].

## Materials and Methods

### Ethics Statement

Primary human monocytes derived macrophages (HMDM) were isolated from buffy coats obtained from healthy adult blood donors at the Blood Transfusion Center, Karolinska University Hospital, Stockholm, Sweden. The donors are approved and covered by insurance according to the regulations at the University Hospital. The identity of the blood donors is unknown to the scientists performing the experiments. Prior to the present study, advice was sought from the Ethical Committee for Human Studies in Stockholm, and a statement was issued that there are no objections to studies of nanomaterials on cells derived from human buffy coats, since the data cannot be traced back to the individual blood donors; hence, no specific ethical permit is required (Decision 2006/3:8) [46]. All the other cell models used in this study are well established cell lines; therefore no ethical permission was required.

### Characterization of nanomaterials

A detailed physico-chemical characterization of the NMs is provided in the JRC Reports [21–23]. Each individual NM in the JRC Repository originates from one batch; an identifying code has been allocated for the material and the single vials have a unique number. The sub-sampling was done under GLP equivalent conditions and each material was homogenized before and during sub-sampling in a special laboratory with cleaned air and solvent-free atmosphere. The NMs used in this study are classified as representative test materials (RTM) and include a (random) sample from one industrial production batch. Generally, the RTM ensures that the particular sample has been homogenized, is sub-sampled into vials under reproducible (GLP) conditions, with the stability of the sub-samples monitored. Thus, to the extent feasible for industrial materials, all the sub-samples from one material should be identical and differences in test results between laboratories for the same end-point should not be attributed to differences in the material tested [21–23].

For the present study the following representative oxide NMs were selected: TiO_2_ (NM-103 and NM-104), ZnO (NM-110 and NM-111) and SiO_2_ (NM-200 and NM-203) (Table A in S1 File). The parameters analysed and the respective methodology applied for characterization of NMs included: composition (wt%) by EDS, surface chemistry by TGA, Gas Chromatography—Mass Spectrometry (GC-MS) and XPS, crystal phase by XRD and DTA, crystalline size (nm) by XRD, primary particle size (nm), particle size distribution number (%), aggregates/agglomerates size (nm) and representative images by TEM, specific surface area (m^2^/g) by BET and SAXS, Zeta potential and IEP (surface charge) by Zetametry and solubility at 24h by SDR.

### Dispersion and endotoxin testing of nanomaterials

For the dispersion of stock concentrations of oxides NMs, the SOP developed under EU NANOGENOTOX Joint Action was used [52]. This method aims to produce a highly dispersed state of NMs by ethanol (EtOH) prewetting to handle hydrophobic materials followed by dispersion in sterile-filtered 0.05% w/v BSA-water. Briefly, the desired stock dispersion was prepared by pre-wetting powder in 0.5 vol% ethanol (96% purity) followed by dispersion in 0.05% BSA-water. The sample was then dispersed by sonication (16 min at 400 W). The described protocol should ensure stable dispersion up to 1h before considering re-dispersing due to onset of agglomeration and/or sedimentation. However, the possible sedimented particles can easily be re-dispersed by vortexing the dispersion for 10 seconds. Long-term storage of NMs in liquids should also be avoided in general due to risk of partial alteration of the original NM or its coating and other hydro-chemical reactions. Following the preparation of stock solutions as indicated above, the working concentrations were prepared in complete culture media and used for the *in vitro* experiments.

Prior to be used for the exposure, the NMs were tested for endotoxin contamination (LPS) using the LAL test (Limulus Amebocyte Lysate Endochrome, Charles River Endosafe, Charleston, SC) according to the manufacturer’s instructions. The importance of endotoxin testing was previously described [24, 25, 53].

### *In vitro* test systems

Twelve different cellular systems representing six different target organs or systems were used in this study (Fig 2).

#### Immune system

Primary human monocytes derived macrophages (HMDM). The peripheral blood mononuclear cells (PBMCs) were isolated from buffy coats from healthy adult blood donors (purchased from Karolinska University Hospital, Stockholm, Sweden) using the Lymphoprep gradient centrifugation and CD14 beads separation method [54]. The whole blood was layered on top of Lymphoprep (Nycomed), a density gradient media, and the tube centrifuged in order to separate the red blood cells, PBMCs and plasma. PBMCs were removed from the tube and washed twice to remove the residues of Lymphoprep and platelets. The cells were then incubated with CD14 MicroBeads (Miltenyi Biotec) in phosphate buffer supplemented with 0.05% BSA and 2 mM EDTA for 60 minutes and passed through a separation column (Miltenyi Biotec) placed in a magnetic field. Freshly isolated monocytes were cultured at 37°C in RPMI-1640 culture medium (Sigma) supplemented with 10% FBS, 2 mM L-glutamine, 100 U/ml penicillin, 100 μg/ml streptomycin and 5 ml of 100 mM sodium pyruvate (Gibco). For differentiation into macrophages, the cells were incubated with 50 ng/ml recombinant macrophage colony-stimulating factor (M-CSF; R&D systems) for 72h. After their differentiation, the cells were washed with PBS and exposed to NMs in RPMI culture medium without FBS for the LDH cytotoxicity assay and supplemented with 10% FBS for the cytokine release evaluation.

Murine peritoneal macrophages (RAW 264.7) used for Resazurin and NRU assays were obtained from the Cell Bank of the Istituto Zooprofilattico Sperimentale della Lombardia ed Emilia-Romagna (Brescia, Italy) and routinely cultured in 10-cm diameter dishes in RPMI 1640 medium supplemented with 10% FBS, streptomycin (100 μg/ml) and penicillin (100 U/ml) in a humidified atmosphere of 5% CO_2_ in air. For the cytotoxicity experiments, cells were seeded on 96-well microplates (Falcon cat. no. 3072) at a density of 3x10^4^ cells/well using complete culture medium supplemented with 10% FBS. The RAW 264.7 cells used for LDH, WST-8 and DCF assays were obtained from ATCC (American Type Culture Collection) and cultured in DMEM with 4.5 g/l D-glucose (Sigma) supplemented with 10% FBS Gold (PanBiotech), 4 mM L-glutamine (Lonza) and 1 mM pyruvate (Lonza).

Murine alveolar macrophages (MH-S), a gift of Prof. Dario Ghigo, University of Torino (Italy), were originally provided by the Cell Bank of the Istituto Zooprofilattico Sperimentale della Lombardia ed Emilia-Romagna (Brescia, Italy). The cells were routinely cultured in 10-cm diameter dishes in RPMI1640 medium supplemented with 10% FBS, 50 μM β-mercaptoethanol, streptomycin (100 μg/ml) and penicillin (100 U/ml) in a humidified atmosphere of 5% CO_2_ in air. For the cytotoxicity experiments, cells were seeded on 96-well microplates (Falcon Cat. No. 3072) at a density of 3x10^4^ cells/well in complete culture medium supplemented with 10% FBS.

#### Respiratory system

Calu-3 cells, derived from a human lung adenocarcinoma (serous cells of proximal bronchial airways) were obtained from the Cell Bank of the Istituto Zooprofilattico Sperimentale della Lombardia ed Emilia-Romagna (Brescia, Italy). Cells were routinely cultured in 10-cm diameter dishes in Eagle’s Minimum Essential Medium (EMEM) supplemented with 1 mM sodium pyruvate, 10% FBS, streptomycin (100 μg/ml) and penicillin (100 U/ml), in a humidified atmosphere of 5% CO_2_ in air. For cytotoxicity experiments, cells were seeded on 96-well plates (Falcon Cat. No. 3072) at a density of 8x10^4^ cells/well. For the barrier integrity experiments, Calu-3 cells were seeded into cell culture inserts with membrane filters (pore size of 0.4 μm, Cat. No. 3095, Becton, Dickinson & Company, Franklin Lakes, NJ, USA), placed into 24-well multitrays, at a density of 7.5x10^4^ cells/well. Before the barrier integrity experiments, cells were allowed to grow until a value of transepithelial electrical resistance (TEER) higher than 1000 Ω*cm^2^ was obtained.

Human bronchial epithelial (16HBE) cells were purchased from the Cell Bank of the Chinese Academy of Medical Sciences (Beijing, China). The cells were cultured in RPMI 1640 medium containing 10% fetal bovine serum (FBS, Gibicol) and 0.1 mg/mL L-glutamine in 5% CO_2_, 37°C atmosphere incubator.

Rat lung epithelial (RLE-6TN) cells obtained from ATCC were cultured in RPMI 1640 medium (Sigma, St Louis, MS, United States) supplemented with 10% FBS Good (PanBiotech, Aidenbach, Germany), 4 mM L-glutamine (Lonza, Basel, Switzerland) and 1 mM pyruvate (Lonza, Basel, Switzerland) under standard cell culture conditions (37°C, 5% CO_2_, humidified).

#### Male reproductive system

TM3 Leydig and TM4 Sertoli mouse cell lines were both purchased from ATCC (ATCC CRL-1714 and ATCC CRL-1715 respectively). These cells were selected for use within this study, as they are traditionally used within the literature for male reproductive toxicity testing. They are semi-immortalized BALB/C mouse cell lines. The two cell types are complementary, having been isolated at same age and using same methodology [55]. Leydig cells are polyhedral cells present within the testicular interstitium. They hold key developmental, reproductive and immune system roles, and are responsible for production of androgens which act on both local and systemic targets. Locally, androgens produced by Leydig cells stimulate Sertoli cells, the ‘nurse’ cells which form the blood-testis barrier and in which spermatogenesis takes place. Both cell lines were cultured within a sterile environment using Dulbecco’s Modified Eagle’s Medium/Nutrient Mixture F-12 Ham 500 ml with L-glutamine, 15 mM 4-(2-Hydroxyethyl)piperazine-1-ethanesulfonic acid (HEPES), and sodium bicarbonate, together with foetal calf serum (FCS) and penicillin/streptomycin. Investigation of normal behaviour in culture and doubling time, together with determination of optimum seeding density within assay plates was undertaken. All assays were replicated at least 5 times, and controlled for passage number (passage 3–9), as work undertaken at commencement of the study showed that TM3 and TM4 cell lines display altered behaviour as they grow ‘older’ in passage. In addition, prior to cytotoxicity testing on TM3 cells, a comparison between normal FCS and charcoal stripped FCS was undertaken (Fig. N in S2 File). Charcoal stripped serum controls for presence of lipophilic materials such as hormones and growth factors which may interfere with endocrine investigations. No difference in viability of cells following exposure to NMs was observed as a consequence of using charcoal stripped (C/S) serum. As a result, all further cell culture work has been undertaken using this serum type at a concentration of 5%.

#### Embryonic cells

Mouse NIH/3T3 cells and D3 mouse Embryonic Stem cells (mES) were purchased from ATCC (Manassas, VA, USA). Mouse NIH3T3 fibroblasts were kept in culture under standard conditions. Briefly, the cells were cultured in Dulbecco's Modified Eagle's Medium with 4.5 g/l D-glucose (DMEM) supplemented with 10% fetal bovine serum, 20 mM Hepes, 2 mM L-glutamine, 50 U/ml penicillin and 50 μg/ml streptomycin (all from Lonza, Basel, Switzerland). Cells were maintained at 37°C in a humidified atmosphere of 5% CO_2_ in air. The NIH/3T3 cell line used for the LDH, WST-8 and DCF assays was obtained from DSMZ and cultured in DMEM (Sigma) supplemented with 10% FBS Good (PanBiotech), 4 mM L-glutamine (Lonza) and 1 mM pyruvate (Lonza) and maintained under the same conditions. D3 cells were routinely cultured under non-differentiating conditions on a feeder layer of γ-irradiated mouse embryonic fibroblasts (MEF) in DMEM with 4.5 g/l D-glucose, supplemented with 15% heat-inactivated fetal calf serum (ES cell tested, Lonza), 20 mM Hepes, 2 mM L-glutamine, 100 μM β-mercaptoethanol, 100 μM non-essential amino acids, 50 U/ml penicillin and 50 μg/ml streptomycin (all from Lonza, Basel, Switzerland) and 103 U/ml Leukemia Inhibitory Factor (LIF, Immunological Sciences, Rome, Italy). For proliferation assays cells were seeded in 96-well plates at a density of 500 cells/well in the presence of different concentrations of the tested NMs. For these experiments D3 cells were cultured in DMEM supplemented with 15% FBS and without LIF (proliferation medium). For differentiation experiments DMEM was supplemented with 20 mM Hepes, 2 mM L-glutamine, 100 μM β-mercaptoethanol, 100 μM non-essential amino acids, 50 U/ml penicillin, 50 μg/ml streptomycin and 10% fetal calf serum (differentiation medium).

#### Gastrointestinal system

The Caco-2 cell line (human epithelial colorectal adenocarcinoma (ATCC collection) was routinely cultured under standard cell culture conditions (37°C, 5% of CO_2_, 90% of humidity) in Dulbecco’s modified Eagle’s medium with high glucose (4.5 g/l), supplemented with 100 U/mL penicillin and 100 μg/mL streptomycin, 4 mM L-glutamine, 1% non-essential amino acids, and 10% heat inactivated foetal bovine serum (FBS). Cells were maintained in culture in 75 cm^2^ flasks. Only cells between passages 45 and 55 were used for subsequent experiments.

#### Kidneys

Normal rat kidney (NRK-52E) cells were obtained from DSMZ (Braunschweig, Germany) and routinely cultured in DMEM (Sigma) with 10% FBS Good (PanBiotech, Aidenbach, Germany), 4 mM L-glutamine and 1 mM pyruvate under standard conditions (37°C, 5% CO_2_, humidified).

### *In vitro* test methods

The toxicity assessment was performed by using ten different assays for cytotoxicity, embryotoxicity, epithelial integrity, cytokine secretion and oxidative stress (Fig 1).

#### Cytotoxicity

Resazurin assay—viable cells reduce the non-fluorescent, membrane permeable, blue compound resazurin into the fluorescent pink dye resorufin, which is extruded into the medium [56]. The reduction takes place only in metabolically active cells. After the exposure to NMs, cells were incubated (1h for RAW 264.7 and MH-S, 2h for Calu-3) with fresh, serum-free medium supplemented with 44 μM resazurin; fluorescence was then measured at 572 nm with a multiplate reader (EnSpire Multimode plate Reader, Perkin Elmer, Waltham, MA, USA). The values of viability were analysed with ANOVA using post hoc Bonferroni test. For the interference evaluation, two distinct approaches were adopted: a) a solution of resazurin in medium, similar to that used for incubating cells in viability assay, was supplemented with NMs at the two highest doses used for experiments (64 and 128 μg/ml) or with vehicle alone (0.05% BSA in water) in the absence of cells. Fluorescence was read every 15 min up to 60 min; b) the incubation with resazurin was performed for 30 min in the presence of cells (RAW 264.7) under the conditions used for viability assay. At the end of the incubation, fluorescence was read. Medium was then supplemented with the indicated NMs, at the two highest doses used for experiments or with vehicle (0.05% BSA solution in water) and the fluorescence was read after 5 min.

Neutral Red Uptake assay (NRU)—this assay provides a quantitative estimation of the number of viable cells in a culture [57]. It is based on the ability of viable cells to incorporate and accumulate the supravital dye neutral red in the lysosomes. After the exposure to NMs, cells were incubated (3h for RAW 264.7 and MH-S, 4h for Calu-3) at 37°C in neutral red solution (0.4% in phosphate buffered saline, PBS). After washing, the dye was extracted with neutral red solubilisation solution (50% ethanol 96%, 49% deionized water, 1% glacial acetic acid). The amount of dye was measured in absorbance at 550 nm with a multiplate reader (EnSpire Multimode plate Reader, Perkin Elmer, Waltham, MA, USA). The values of viability were analyzed with ANOVA using post hoc followed by Bonferroni test. For the *interference evaluation*, the neutral red solution (in culture medium without phenol red) was supplemented with NMs (at 20, 40 and 80 mg/cm^2^) or with the vehicle alone (0.05% BSA in water). After 30 min of incubation the mixture was centrifuged at 300*g* for 3 min, the supernatants resuspended in ethanol/acetic acid and the OD was measured.

WST-1 assay—TM3 Leydig (5x10^4^ cells/well) and TM4 Sertoli (1x10^4^ cells/well) were seeded in microtiter plates. NM stock solutions (1 mg/ml) were prepared and sonicated for 16 min. Stock solutions were diluted to 200 μg/ml with further dilutions made thereof down to 0. In addition the following ZnO NM, dry powder was wetted with 0.5% ethanol prior to sonication in 2% FCS deionized and autoclaved water. 6 mg of coated ZnO was weighed out each time and 30 μl of 0.5% ethanol was added drop by drop and the powder mixed by turning the tube around in the hand. 5.970 ml of 2% FCS water was then added and sonicated in the usual manner. Cells were exposed to NM for a period of 24h. After the incubation period, the old medium was discarded from the wells and rinsed with media before adding fresh culture medium with a final volume of 100 μl per well. Then, 10 μl of WST-1, cell proliferation reagent, was added to each well. The microtiter plate was then incubated again for 2h at 37°C with 5% CO_2_. After the incubation period, the cell viability was measured using a plate reader at 450 nm. The experiment was carried out using six replicates. Statistical analysis was conducted using MINITAB version 15.1 software and data was checked for normality before undergoing analysis by way of ANOVA followed by Tukey’s post-hoc test.

WST-8 assay on 16HBE cells was performed using the Cell Counting Kit (CCK-8) (Dojindo Laboratories, Japan). The cells were seeded at a density of 8×10^3^ cells per well in 96-well plates in medium and incubated for 24h. Then, the medium was replaced with 100 μl of fresh medium containing different concentrations of NMs. After incubation for 24h at 37°C, reagents were added according to the manufacturer’s protocol. Absorbance was recorded at 450 nm by an Infinite M200 microplate reader (Tecan, Durham, USA). The mean absorbance of non-exposed cells was taken as the reference value (100% cellular viability).

WST-8 assay on RAW 264.7, RLE-6TN, NRK-52E and NIH/3T3 was performed slightly differently without using the CCK-8 kit. Cells were seeded for 24h in 96-well plates at a density of 5x10^3^ cells per well (RAW 264.7: 1.5x10^4^cells per well) and afterwards exposed to NMs for 24h. Triton X-100 (0.01%), diluted in DMEM cell culture media supplemented with 25 mM HEPES (Lonza, Basel, Switzerland) was used as positive control for total cell lysis. Cells were washed with DMEM prior to incubation with 100 μl WST8-working medium (0.7 mM WST-8 (GeneScript, Piscataway, NJ, USA), 25 μM 1 methoxy-phenazine methosulphate (PMS) (Applichem, Darmstadt, Germany) in DMEM supplemented with 25 mM HEPES) for 45 min and washed again. The absorbance was measured at 450 nm (absorption of WST8 formazan) and 620 nm (turbidity correction) by the FLUOstar reader (BMG Labtech GmbH, Offenburg, Germany). Each experiment was repeated at least 3 times with four replications. The results were calculated as % viability means (+/- SD) of media control, cells without nanomaterials. Statistical analysis was performed using one-way ANOVA followed by Tukey’s *post hoc* test.

Lactate dehydrogenase assay (LDH) in HMDM was performed using the CytoTox 96 non-radioactive cytotoxicity kit (Promega G1780) as previously reported [54]. After the exposure period, 50 μl of culture supernatant were removed from the cells and loaded to a 96 well plate. For the measurement of intracellular LDH, the cells were exposed to lysis buffer at 37°C for 30 min. 50 μl of the lysis were transferred to a 96 well plate and 100 μl of the reaction substrate were added to each sample. The formation of red formazan was read at 492 nm using a spectrophotometer (Tecan Infinite 200). The percentage of cell viability was calculated based on the ratio between the absorbance of each sample compared with the negative control. Results of LDH assay were expressed as % cell viability mean (± SD), from at least three independent healthy blood donors (primary HMDM). Statistical analysis was performed using one-way ANOVA followed by Tukey’s *post-hoc* test.

Similarly, the LDH assay on RAW 264.7, RLE-6TN, NRK-52E and NIH/3T3 cells was performed as previously reported [11]. Briefly, cells were seeded in 96-well plates at a density of 3x10^4^ cells per well (*RAW 264*.*7*: 6x10^4^cells per well). After 24h, cells were exposed to NMs for 24h, Triton X-100 (0.01%) diluted in DMEM cell culture media supplemented with 25 mM HEPES (Lonza, Basel, Switzerland) was used as positive control for total cell lysis. Subsequently, 50 μl from the supernatant was transferred to a new plate and mixed with 100 μL assay solution (0.66 mM INT (Sigma, St. Louis, MO, United States), 0.28 mM phenazine methosulphate (PMS) (Sigma), 1.3 mM nicotinamide adenine dinucleotide (NAD) (Sigma), 56 mM lactate (Sigma) in 175 mM Tris(hydroxymethyl)-aminomethan (TRIS) (Roth, Karlsruhe, Germany), pH 8.2). The formation of INT formazan was monitored at 492 nm for 30 min at 28°C using the FLUOstar or NOVOstar spectrophotometers (BMG Labtech GmbH, Offenburg, Germany). Each experiment was repeated at least 3 times with four replicates. The results were calculated as % INT light absorption means (± SE) of media control, cells without nanomaterials, which were set at 100%. Statistical analysis was performed using one-way ANOVA followed by Tukey’s *post hoc* test.

Colony Forming Efficiency (CFE) assay was performed to assess the cytotoxic and cytostatic potential of NMs, both after short exposure (72h) and after long-term, repeated dose exposure (72h + 96h + 72h = 10 days). Briefly, on Day 1, 200 Caco-2 cells were seeded in 3 ml of fresh complete medium in each 60-mm Petri dish (six replicates per concentration). After 24h of incubation, NMs were added to the cells. All NMs were used at the concentration of 100 μg/ml. A solvent control (0.05% BSA) and positive control, Na_2_CrO_4_·6H_2_O, was run in parallel. The concentration of Na_2_CrO_4_·6H_2_O were 100 μM for the short-term and 50 μM for the long-term, repeated dose exposure. In the case of short, acute exposure the cells were kept in contact with NMs for 72h; then, on Day 4 medium was removed and replaced with complete fresh medium, and the cells were cultured for additional 7 days. For long-term, repeated dose treatments, on Day 4 and Day 8 the treatment suspensions were replaced with fresh suspensions of NMs in complete medium. At the end of each treatment (Day 11 after seeding), the medium was removed, the colonies of Caco-2 cells were first fixed using a solution of 4% (v/v) formaldehyde in PBS, then stained using 0.4% (v/v) of Giemsa in MilliQ water. After drying, colonies (composed of at least 50 cells) were counted using an automated cell colony counter (GelCount; Oxford Optronix Ltd., Oxford, UK). In addition, the area of the colonies was determined. The results were normalized to the control (cells exposed to fresh complete culture media containing the solvent, 0.05% BSA, undergoing the same number of medium changes) and expressed as:

$$\%CFE=\frac{average\;number\;of\;colonies\;in\;treatment}{average\;number\;of\;colonies\;in\;solvent\;control}\times 100$$ (1)

where a reduction of the number of colonies formed in the treatment, with respect to the solvent control, is a measure of cytotoxicity.

$$\%Average\;colony\;area=\frac{average\;area\;of\;colonies\;in\;treatment}{average\;area\;of\;colonies\;in\;solvent\;control}\times 100$$ (2)

where a decrease in average colony area is an indicator of cytostatic effects.

Two independent experiments were performed, with six replicates in each experimental run. The corresponding Standard Error of the Mean was calculated. The statistically significant difference for CFE values versus controls was calculated by the one-way ANOVA analysis, followed by Dunnet’s post-test.

#### *In vitro* embryotoxicity

Embryonic Stem Cell Test (EST)—in order to assess the embryotoxic potential of oxide NMs under study, the Embryonic Stem Cell Test (EST) was applied as previously described [26]. The proliferation and differentiation assays were used to obtain three endpoints: i) the concentration of NM reducing by 50% the differentiation of mES cells into contracting myocardial cells (ID_50_), ii) the concentration of NM reducing by 50% the proliferation of mES (IC_50_ES) and iii) the concentration of NM reducing by 50% the viability of NIH3T3 cells (IC_50_3T3). The three values are then integrated in an algorithm that allows classifying the NMs as strong-, weak- and non-embryotoxic, as described before [26, 58].

In the proliferation experiments, following the EST guideline, proliferation medium containing the material under analysis was changed on day 3 and 5 of culture. On day 10, cell proliferation was evaluated by the colorimetric assay, WST-1 (Cell Proliferation Reagent, Roche). After removing the culture medium from the 96-well plate, and a washing step in PBS, 100 μl of culture medium and 10 μl of the WST-1 reagent were added to each well. Plates were incubated for 2h at 37°C in a humidified atmosphere of 5% CO_2_, and then shaken for 1 min. Absorbance was measured at 450 nm (reference wavelength at 655 nm) using a microplate reader (Bio Rad Microplate Reader 3550). Data are shown as mean ± standard error of at least 15 values from 4 independent experiments, and presented as percentages relative to control.

For mES cell differentiation into embryoid bodies (EBs) the “hanging drops” protocol was applied according to the EST. Eight hundred cells were suspended in 20 μl of differentiation medium in the presence of the different NMs at the same concentrations used for the proliferation assay, and cultured for three days. EBs were transferred to fresh test medium in a 6 cm diameter Petri dish and cultured for two additional days, and then transferred to a 24-well plate in test medium (one EB/well). After 5 days, the presence of contracting cardiomyocytes was evaluated under the microscope. The IC_50_ and ID_50_ values obtained with these procedures were introduced in the following algorithm:

$$\begin{array}{cc} & \mathrm{Function}\;I:5.92\mathrm{Ig}({\mathrm{IC}}_{503T3})+3.50\mathrm{Ig}({\mathrm{IC}}_{50D3})-5.31({\mathrm{IC}}_{503T3}-{\mathrm{ID}}_{50}∕{\mathrm{IC}}_{503T3})-15.7\\ & \mathrm{Function}\;\mathrm{II}:3.65\mathrm{Ig}({\mathrm{IC}}_{503T3})+2.39\mathrm{Ig}({\mathrm{IC}}_{50D3})-2.03({\mathrm{IC}}_{503T3}-{\mathrm{ID}}_{50}∕{\mathrm{IC}}_{503T3})-6.85\\ & \mathrm{Function}\;\mathrm{III}:-0.125\mathrm{Ig}({\mathrm{IC}}_{503T3})-1.92\mathrm{Ig}({\mathrm{IC}}_{50D3})+1.50({\mathrm{IC}}_{503T3}-{\mathrm{ID}}_{50}∕{\mathrm{IC}}_{503T3})-2.67 \end{array}$$

If I > II and I > III the NM is defined as non-embryotoxic, if II > I and II > III as weakly-embryotoxic and if III > I and III > II as strongly-embryotoxic.

#### Epithelial barrier integrity

Transepithelial Electrical Resistance (TEER) on Calu-3 cells—for measurements of transepithelial electrical resistance, an indicator of epithelial cell barrier integrity, Calu-3 cells were seeded into cell culture inserts with membrane filters (pore size of 0.4 μm) for Falcon 24-well-multitrays (Cat. No. 3095, Becton, Dickinson&Company, Franklin Lakes, NJ, USA) at a density of 7.5x10^4^ cells/insert. Cells were allowed to grow, usually for 10–14 days, until a value of TEER higher than 1000 Ω*cm^2^ was reached, indicating the formation of a tight epithelial monolayer. TEER measurements were made using an epithelial volt-ohm-meter (EVOM, World Precision Instruments Inc., Sarasota, FL, USA). NMs were added in the apical chamber from the stock solutions without changing the medium. TEER changes were expressed as the percentage of the initial value adjusted for control cell layers according to the equation:

$$TEER\;\left(\%\right)=\;\frac{final\;TEER_{treated}}{final\;TEER_{control}}\times \frac{initial\;TEER_{control}}{initial\;TEER_{treated}}\times 100$$

The values of TEER were analyzed with ANOVA followed by post hoc Bonferroni test.

Transepithelial Electrical Resistance (TEER) on Caco-2 cells—the effects of the NMs on the intestinal barrier were assessed in Caco-2 cells differentiated on cell culture inserts. In these experiments, the changes in Trans-Epithelial Electrical Resistance (TEER) were monitored for 21 days, to assess the epithelial barrier integrity after a long-term, repeated-dose exposure to NMs.

To prepare the cultures on inserts, 7.5x10^4^ cells in 2.5 ml fresh complete medium were seeded in the apical compartment of the 6-well plate inserts (membrane pore diameter 0.4 μm, surface 4.2 cm^2^; BD, Italy). After seeding Caco-2 cells were cultured in complete medium for 21 days during which they differentiate spontaneously into polarized intestinal cells. Every 3 or 4 days the culture medium was refreshed and TEER was measured to check barrier formation. After three weeks of culture, when the epithelial barrier was well formed (stable, high TEER values), the medium in the apical compartment (2.5 ml) was replaced with treatment medium containing NMs at the concentration of 100 μg/ml, while the medium in the basolateral compartment was replaced with fresh one (2.5 ml). The treatment medium was changed every 3 days. Each experiment included a negative control (cells in culture medium), a positive control (cells exposed to a Na_2_CrO_4_ · 6H_2_O; 50 μM) and a solvent control (0.05% BSA). TEER measurements were taken every 3 days immediately before the NMs addition by the Millicell-ERS system (MILLIPORE, Italy). TEER values were calculated using the following equation:

$$TEER\;\left(\Omega \cdot \text{cm}²\right)=\left(R_{total}-R_{blank}\right)\times A$$

where *R*_*total*_ is the resistance measured, *R*_*blank*_ is the resistance of control filters without cells and *A* is surface of the filter (4.2 cm^2^).

TEER was monitored for 21 days. This protocol represents the *in vitro* long-term exposure approach.

#### Cytokine release

TNF-α released by primary human macrophages in the culture medium was measured using ELISA kit following the instructions provided by the manufacturer (MABTECH, 3510-1A-20). The absorbance of the reaction product was measured at 405 nm using a spectrophotometer (Tecan Infinite 200) and the results for each sample were calculated using a standard curve of recombinant human TNF-α protein. LPS (100 ng/ml; Sigma Aldrich, L4391) was used as positive control for TNF-α release. Prior to the cytokine measurements, a LAL test (ENDOCHROME Assay, Charles River Endosafe) was performed in order to evaluate the endotoxin (LPS) contamination of the NMs. Also, the possible interferences between the NMs and the assay reagent and readings were evaluated.

#### Oxidative stress

Dichlorofluorescein assay (DCF) in 16HBE cells used CM-H_2_DCFDA and Hoechst 33342 to stain the intracellular and nuclear ROS after 24h treatment of the cells. The average cellular fluorescence intensity by DCF staining was quantified according to the cell number by the measurement of IN cell analyzer 2000 (GE Healthcare, USA). In case of RAW 264.7, RLE-6TN, NRK-52E and NIH/3T3, the DCF assay was performed as previously reported [11]. Briefly, cells were seeded in 96-well plates at a density of 3x10^4^ cells per well (RAW 264.7: 6x10^4^cells per well). After 24h, cells were exposed to NM dispersions for 1h. Carbon black (10 μg/cm^2^; Carbon black pigment, Degussa GmbH, Essen, Germany) was previously described to induce oxidative stress [11] and was therefore used as positive control. Cells were washed twice with Krebs-Ringer Buffer (KRB) and afterwards incubated with 2’,7’-dichlorodihydrofluorescein diacetate (H_2_DCF-DA) working solution (5 μM in KRB) for 45 min. To remove excess H_2_DCF-DA, cells were washed again twice before monitoring the fluorescence in a spectrophotometer (Excitation 485 nm, emission 520 nm; FLUOstar, BMG Labtech GmbH, Offenburg, Germany). Each experiment was repeated at least 3 times with six replications each, and results were illustrated as % ROS activity means (± SE) of positive control, cells without nanomaterials, which was set as 100% ROS activity. Statistical analysis was performed using one way ANOVA followed by Tukey’s *post hoc* test.

## Supporting Information

S1 File Table A—Physico-chemical parameters of representative oxide NMs used for the in vitro studies. (PDF)

S2 File Figures A-N—*in vitro* toxicity testing of oxide nanomaterials. (PDF)

S3 File Figures O-U—evaluation of oxide nanomaterials interferences with LDH, ELISA, Resazurin, NRU and WST-8 and WST-1 assays. (PDF)
