# Supplementary material for: Comprehensive In Vitro Toxicity Testing of a Panel of Representative Oxide Nanomaterials: First Steps towards an Intelligent Testing Strategy
Source: PLoS One. 2015 May 21;10(5):e0127174. doi: 10.1371/journal.pone.0127174 (PMC4440714; doi:10.1371/journal.pone.0127174)
Supplement: S1 File — (PDF) [file pone.0127174.s001.pdf]

Supporting Information – S1 File

Table A. Physico-chemical parameters of representative oxide NMs (TiO_2, ZnO and SiO_2) used for the *in vitro* studies [21-23]

TiO_2	Composition (wt%)	Surface chemistry	Crystal phase	Crystalline size (nm)	Primary Particle size (nm)	Particle size distribution Number (expressed in %)	Aggregates/ agglomerates size (nm)	Specific surface area (m^2/g)	Zeta potential and IEP (surface charge)	Solubility	TEM images
**NM-103**	Ti 54.74<br>O 40.82<br>Al 3.43<br>Si 0.68<br>Fe 0.06<br>**(EDS)**	2% mass loss **(TGA).**<br>dimethoxydimethyl-silane, silanes (2 wt%)<br>**(GC-MS);**<br>O: 56, C: 25.9, Ti: 10.7, Al: 4.9 , (Fe, Ca: 2.5); at %<br>**(XPS)**	Rutile **(XRD)**	19→28 **(XRD)**	24.7±2.3 **(TEM)**	<100 nm: 51.8%, <50 nm: 12.7 %, <10 nm: 0.1% **(TEM)**	140, fractal dimension Df: 2.2 (SAXS) Morphology: Low sphericity, very angular to sub-angular **(TEM)**	51 **(BET),** 51.1 **(SAXS)**	DI water + buffer:<br>at pH< 4 30 mV;<br>at pH > 8.2 -40mV;<br>IEP: 8.2 (**Zetametry**)	Al, Si detected in 0.05% BSA+water, DMEM, Gamble's solution; NM-103 only in DMEM **(24 h SDR)**	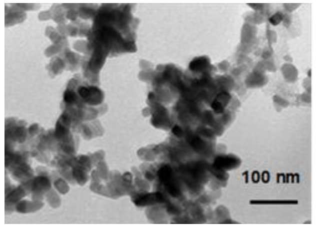
**NM-104**	Ti 55.60<br>O 59.81<br>Al 3.22<br>Si 0.18<br>**(EDS)**	2% mass loss **(TGA).**<br>Tetramethyl silicate, Glycerol; Silane; Hexadecanoic acid methyl ester; Hexadecanoic acid; Octadecanoic acid<br>**(GC-MS);**<br>O: 63.5, C: 16.3, Ti: 13.1, Al: 7.1; at %<br>**(XPS)**	Rutile **(XRD)** Phase transition at 323ºC **(DTA).**	18→29 **(XRD)**	25.0±1.7 **(TEM)**	<100 nm: 53.3%, <50 nm: 12.1%, <10 nm:0.1% **(TEM)**	160, fractal dimension Df: 2.3 **(SAXS).** Morphology: Low sphericity, angular to sub-rounded **(TEM)**	56 **(BET)**, 52.2 **(SAXS)**	DI water + buffer:<br>at pH< 4 30 mV;<br>at pH > 8.2 -40mV;<br>IEP: 8.2 (**Zetametry**)	Al detected in 0.05% BSA+water, DMEM, Gamble's solution; NM-104 only in DMEM **(24h SDR)**	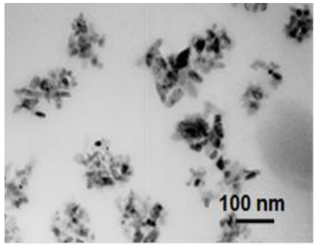

ZnO	Composition (wt%)	Surface chemistry	Crystal phase	Crystalline size (nm)	Primary Particle size (nm)	Particle size distribution Number (expressed in %)	Aggregates/ agglomerates size (nm)	Specific surface area (m^2/g)	Zeta potential and IEP (surface charge)	Solubility	TEM images
**NM-110**	Zn 89.90<br>Ni 0.0009<br>Pb 0.0008<br>Co 0.0003<br>Si 0.0195<br>Al 0.024<br>Ca 0.044<br>**(ICP-OES)**	No weight loss detected<br>**(TGA)**<br><br>**Uncoated.**<br>C:69, Ce:0.0, O:25.1, Si:0.0, Zn:5.6; at %<br>**(XPS)**	Hexagonal zincite structure<br><br>**(XRD)**	41.5<br><br>**(XRD)**	Two main types of morphology:<br><br>1) 20-250 nm , aspect ratio ~1<br>2) 50-350 nm, aspect ratio 2 - 7.5.<br>Mean diameter ±SD: 158±158<br>Mean aspect ratio±SD: 1.7 ±0.6<br>**(TEM)**<br><br>151 ± 55.6 (Feret's mean diameter ± 1 SD) **(SEM)**	Relatively high number of small-size<br><br>Particles, peak-mode around 30 - 40 nm another major broad size mode ca. 200 nm. Merged broad bimodal or clearly bi- or tri-modal in the µm-range<br>**(FMPS/APS)**	275±4 with PDI: 0.145 (in DI water)<br>**(DLS)**<br><br>193±3 (in DI water), 309±10 (in seawater)<br>**(CPS)**	12.4±0.6<br><br>(**BET)**	In DI water: 24.3±0.4 mV<br><br>In DI water +5mM NaCl: 20.8±0.8 mV<br>**(Zetasizer)**	2.5 – 4 ppm in DI water after 6-21 days<br><br>**(Colorimetric test)**	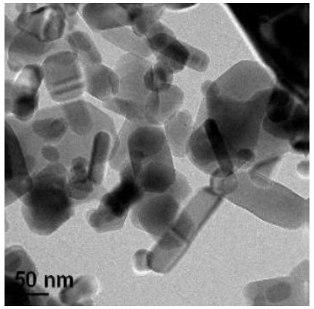
**NM-111**	Zn 87.39<br>Ni 0.0009<br>Pb 0.0008<br>Co 0.0003<br>Si 0.0265<br>Al 0.027<br>Ca 0.074<br>**(ICP-OES)**	1% mass loss<br>**(TGA)**<br><br>**Coated** with triethoxycapryl silane.<br>C:67.9, Ce:0.0, O:24.3. Si:3.5, Zn:4.3; at %<br>**(XPS)**	Hexagonal zincite structure<br><br>**(XRD)**	33.8<br><br>**(XRD)**	Two main types of morphology:<br>1) 20-200 nm, aspect ratio ~1<br><br>2) 10-450 nm, aspect ratio 2 - 8.5.<br>Mean diameter ±SD: 152±111<br>Mean aspect ratio±SD: 1.8 ±0.7<br>**(TEM)**<br><br>140.8 ± 65.8 (Feret's mean diameter ± 1 SD) **(SEM)**	Relatively high number of small-size<br><br>Particles, peak-mode around 30 - 40 nm another major broad size mode ca. 200 nm. Merged broad bimodal or clearly bi- or tri-modal in the µm-range<br>**(FMPS/APS)**	253±1 with PDI: 0.401 (in DI water)<br>**(DLS)**	15.1±0.6<br>**(BET)**	-	-	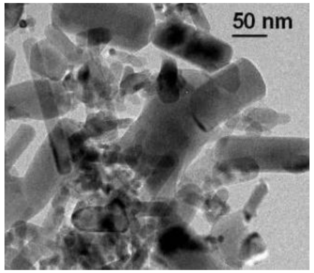

SiO_2	Composition (wt%)	Surface chemistry	Crystal phase	Crystalline size (nm)	Primary Particle size (nm)	Particle size distribution Number (expressed in %)	Aggregates/ agglomerates size (nm)	Specific surface area (m^2/g)	Zeta potential and IEP (surface charge)	Solubility	TEM images
**NM-200**	Si 44.77<br>O 53.02<br>Al 0.46<br>Na 0.88<br>S 0.87, traces of Fe, K<br>Mg, Zr<br>**(ICP-OES)**	4% mass loss<br>**(TGA)**<br>O: 70.8,<br>Si: 24.1 ,<br>C:4.1 ,<br>Na:1 ,<br>S:0.06 ; at % **(XPS)**	Synthetic amorphous silica; crystalline impurities of Na_2SO_4<br>**(XRD)**	amorphous	14 ± 7<br>**(TEM)**	<100 nm - 88.7%, <50 nm - 69.8%<br><10 nm - 1.7%<br>**(TEM)**	440, fractal dimension Df: 2.45<br>**(SAXS)**<br>200 **(DLS)**,<br>Morphology: Sub-rounded, low to medium sphericity<br>**(TEM)**	189.16<br>**(BET),**<br>123.3<br>**(SAXS)**	In DI water:<br>-47.5 mV<br>IEP <2<br><br>In DI water +0.05 % BSA:<br>-18 mV<br><br>**(Zetametry).**	Al and NM-200 detected in 0.05% BSA+water, DMEM, Gamble's solution<br>**(24 h SDR)**	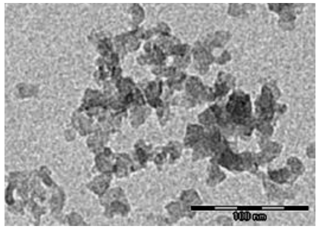
**NM-203**	Si 46.32<br><br>O 53.21<br>Al 0.43<br>S 0.04<br>**(EDS)**	No mass loss<br>**(TGA)**<br>O: 71.7,<br>Si: 26, C: 2.3; at%<br>**(XPS)**	Synthetic amorphous silica<br>**(XRD)**	amorphous	13± 6<br>**(TEM)**	<100 nm - 77.5%, <50 nm - 48.4%<br><10 nm - 0.3%<br><br>**(TEM)**	Aggregates with complex open structure.<br>Morphology: Low sphericity, angular.<br>**(TEM)**<br>Polydisperse material<br>**(DLS)**	203.92<br>**(BET),**<br>167.2<br>**(SAXS)**	In DI water:<br>-46.1 mV<br>IEP <2<br><br>In DI water +0.05 % BSA:<br>-18 mV<br>**(Zetametry)**	Al and NM-203 detected in 0.05% BSA+water, DMEM, Gamble's solution<br><br>**(24 h SDR)**	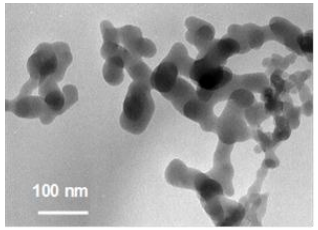

Abbreviations: CPS – Centrifugal Particle Sedimentation, DLS - Dynamic Light Scattering, DTA - Differential Thermal Analysis, EDS - Energy-Dispersive X-ray Spectroscopy, GC-MS - Gas Chromatography–Mass Spectrometry, ICP-OES - Inductively Coupled Plasma Optical Emission Spectrometry, IEP - Iso-Electric Point, SAXS - Small Angle X-ray Scattering, SEM - Scanning Electron Microscopy, SDR - Sensor Disk Reader, SiO_2 - silicon dioxide, TGA - thermogravimetric analysis, TiO_2 – titanium dioxide, TEM - Transmission Electron Microscopy, ZnO – zinc oxide, XRD - X-ray Diffraction.
