# Supplementary material for: Comprehensive In Vitro Toxicity Testing of a Panel of Representative Oxide Nanomaterials: First Steps towards an Intelligent Testing Strategy
Source: PLoS One. 2015 May 21;10(5):e0127174. doi: 10.1371/journal.pone.0127174 (PMC4440714; doi:10.1371/journal.pone.0127174)
Supplement: S2 File — (PDF) [file pone.0127174.s002.pdf]

Supporting Information – S2 File

Supplementary figures: *in vitro* toxicity testing of oxide nanomaterials

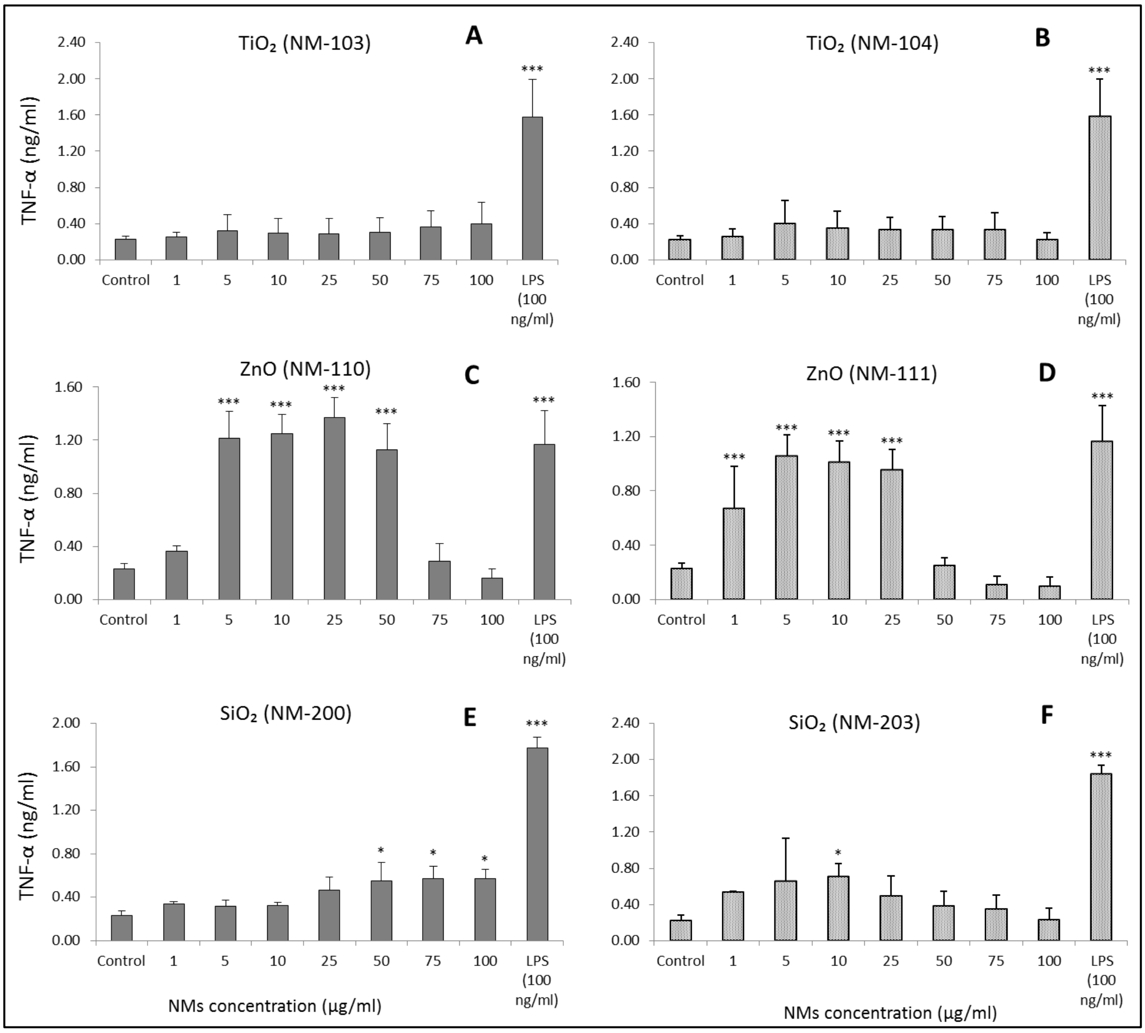

Fig. A. Release of pro-inflammatory cytokine TNF-α in the culture medium by HMDM after 24h exposure to TiO_2 NM-103 (A) and NM-104 (B), ZnO NM-110 (C) and NM-111 (D) and SiO_2 NM-200 (E) and NM-203 (F)

Results are expressed as TNF-α release (ng/ml) (mean ± SD) analyzed in triplicates from three or four independent healthy blood donors (HMDM) analyzed by ELISA. Statistical analysis was performed using one-way ANOVA followed by Tukey's post-hoc test (***$p < 0.001$). The LAL test performed for all six nanomaterials showed LPS levels below 0.5 EU/ml.

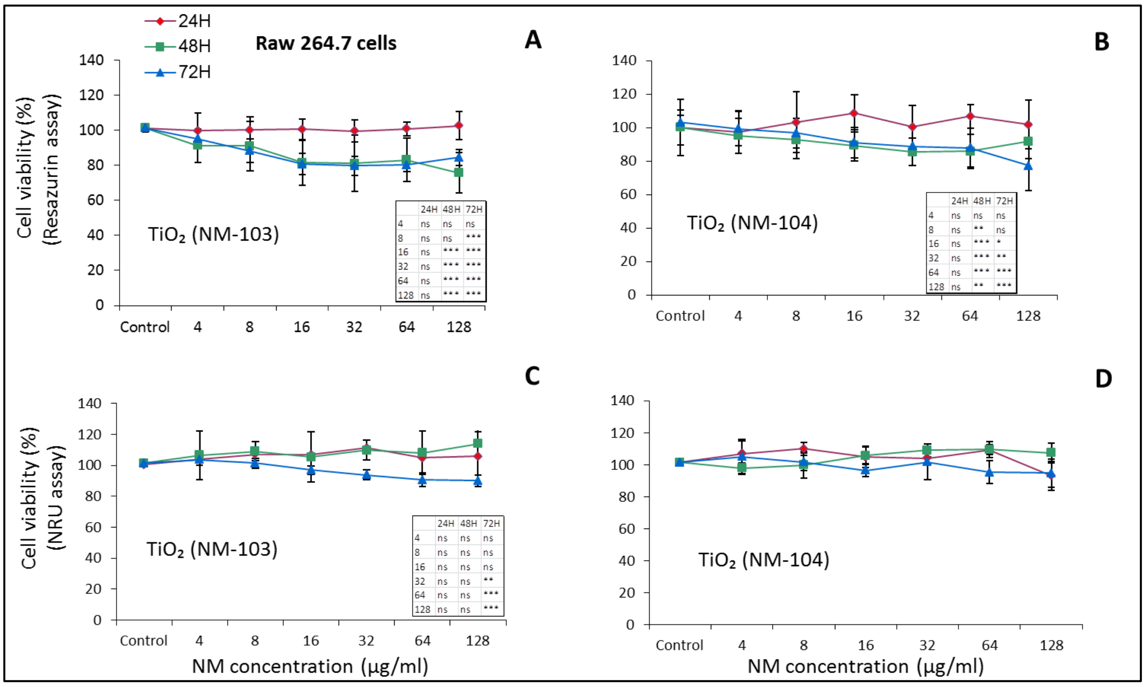

Fig. B. The effects of TiO2 NM-103 and NM-104 on the viability of RAW 264.7 cells evaluated by Resazurin (A and B) and NRU (C and D) assays

Comparative evaluation of cell viability by resazurin assay (A and B) and NRU assay (C and D) after exposure to TiO2 (NM-103 and NM-104). Cells were grown for 24h in complete growth medium and then exposed for 24h, 48h and 72h to different concentrations of TiO2 NMs. At the end of the incubation, cell viability was assessed using two different assays (resazurin and NRU). Data are means ± SD of 10 independent determinations in two separate experiments. Statistical analysis was performed using one-way ANOVA followed by Bonferroni post-hoc test. *$p < 0.05$, **$p < 0.01$, ***$p < 0.001$.

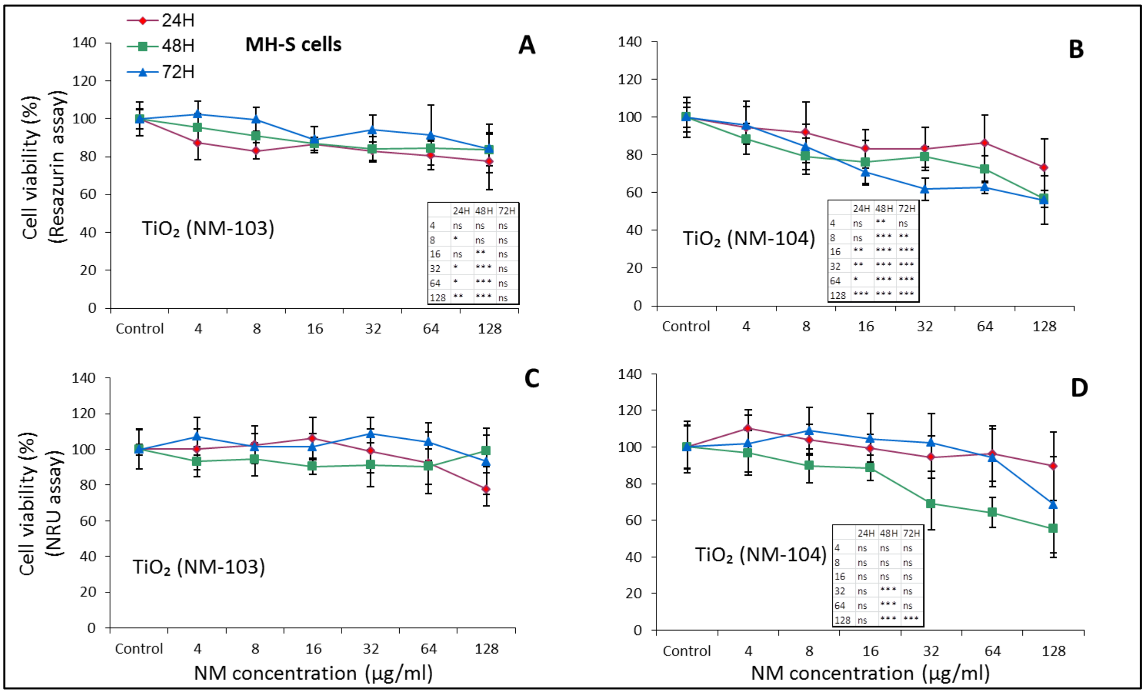

Fig. C. The effects of TiO2 NM-103 and NM-104 on the viability of MH-S cells evaluated by Resazurin (A and B) and NRU (C and D) assays

Comparative evaluation of cell viability by resazurin assay (A and B) and NRU assay (C and D) after exposure to TiO2 (NM-103 and NM-104). Cells were grown for 24h in complete growth medium and then exposed for 24h, 48h and 72h to different concentrations of TiO2 NMs. At the end of the incubation, cell viability was assessed using two different assays (resazurin and NRU). Data are means ± SD of 10 independent determinations in two separate experiments. Statistical analysis was performed using one-way ANOVA followed by Bonferroni post-hoc test. *p< 0.05, **p< 0.01, ***p< 0.001.

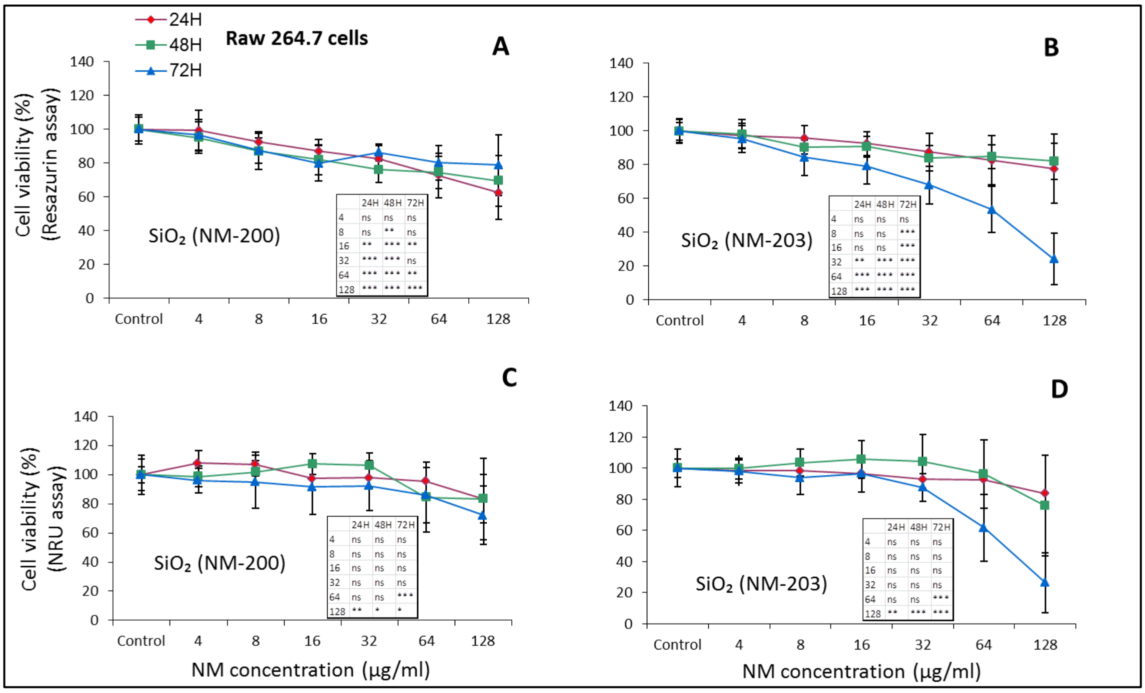

Fig. D. The effects of SiO2 (NM-200 and NM-203) on the viability of RAW 264.7 cells evaluated by Resazurin (A and B) and NRU (C and D) assays

Comparative evaluation of cell viability by resazurin assay (A and B) and NRU assay (C and D) after exposure to SiO2 (NM-200 and NM-203). Cells were grown for 24h in complete growth medium and then exposed for 24h, 48h and 72h to different concentrations of SiO2 NMs. At the end of the incubation, cell viability was assessed using two different assays (resazurin and NRU). Data are means ± SD of 10 independent determinations in two separate experiments. Statistical analysis was performed using one-way ANOVA followed by Bonferroni post-hoc test. *$p < 0.05$, **$p < 0.01$, ***$p < 0.001$.

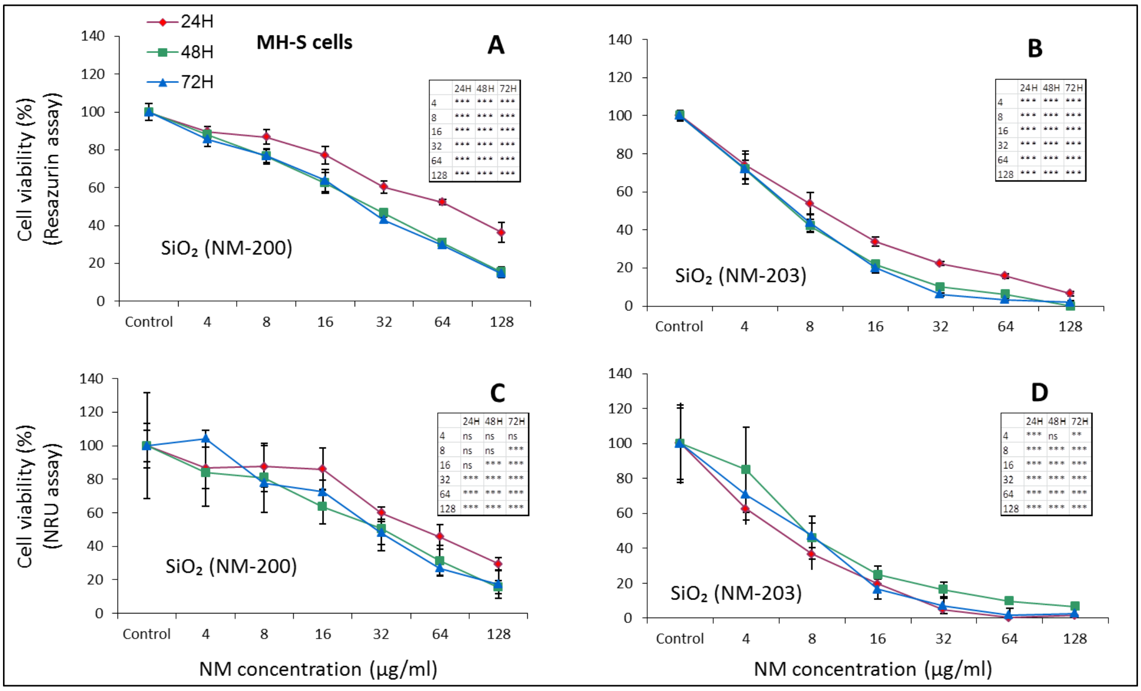

Fig. E. The effects of SiO2 (NM-200 and NM-203) on the viability of MH-S cells evaluated by Resazurin (A and B) and NRU (C and D) assays

Comparative evaluation of cell viability by resazurin assay (A and B) and NRU assay (C and D) after exposure to SiO2 (NM-200 and NM-203). Cells were grown for 24h in complete growth medium and then exposed for 24h, 48h and 72h to different concentrations of SiO2 NMs. At the end of the incubation, cell viability was assessed using two different assays (resazurin and NRU). Data are means ± SD of 10 independent determinations in two separate experiments. Statistical analysis was performed using one-way ANOVA followed by Bonferroni post-hoc test. **p< 0.01, ***p< 0.001.

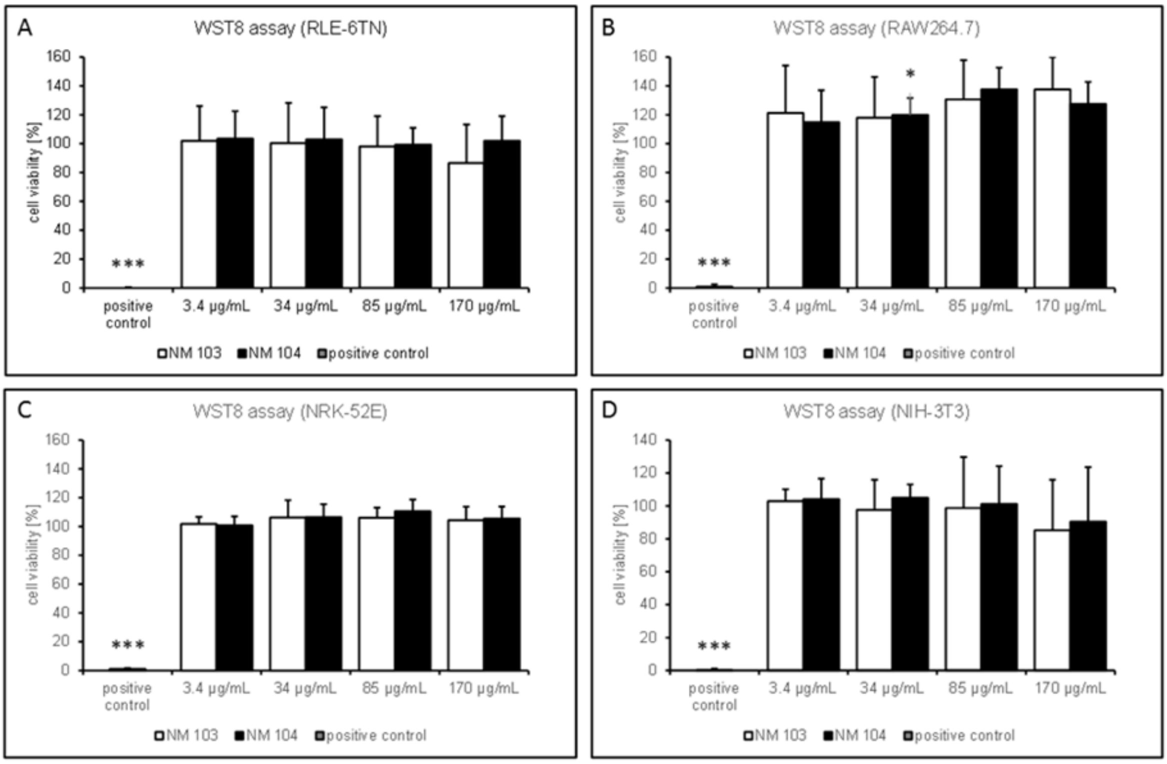

Fig. F. Effects of NM-103 and NM-104 on cell viability of four different cell lines: RLE-6TN (A), RAW264.7 (B), NRK-52E (C) and NIH-3T3 (D)

Cell viability was determined by the WST8 assay after exposure of four different cell lines, RLE-6TN (lung epithelial cells), RAW 264.7 (macrophages), NRK-52E (kidney cells) and NIH-3T3 (embryonic cells) to NM-103 or NM-104 for 24h. Results are expressed as % of medium control, cells without nanomaterials, which was set at 100% cell viability and means (± SD) were obtained from at least four independent experiments with four replicates each. Statistical analysis was performed using one way ANOVA followed by Tukey's *post hoc* test (*$p < 0.05$; ***$p < 0.001$). Viability of cells exposed to 0.01% Triton X-100 serving as a positive control for total cell lysis was near zero in all experiments. For all cell lines no significant concentration dependent decrease of cell viability was detectable, for NM-103 and NM-104. Only in case of RAW 264.7, a low significant increase of OD values was observed for one particle concentration (34 µg/ml, $p < 0.05$), most likely due to the intensive uptake activity of RAW264.7 and high optical activity of TiO_2.

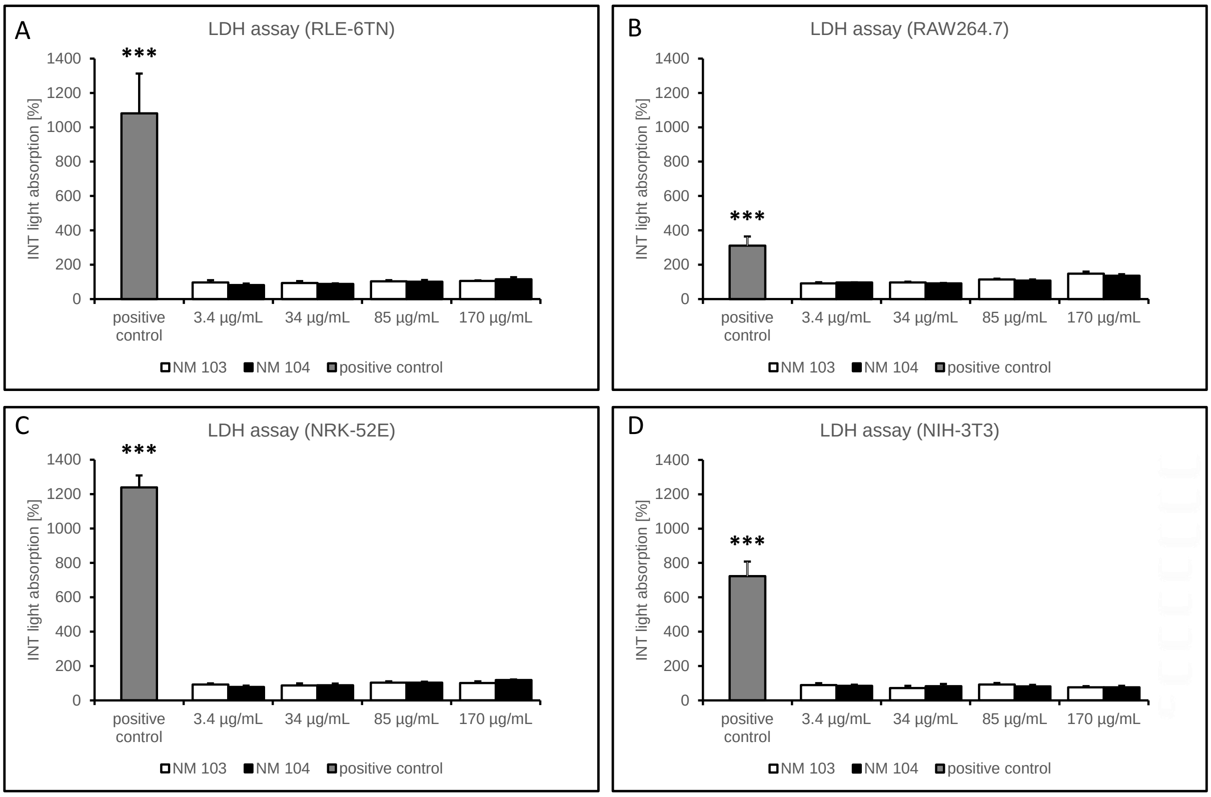

Fig. G. Effects of NM 103 and NM 104 on cell death of four different cell lines: RLE-6TN (A), RAW 264.7 (B), NRK-52E (C) and NIH-3T3 (D)

Cell death was determined by LDH release of four cell lines: RLE-6TN (lung epithelial cells), RAW 264.7 (macrophages), NRK-52E (kidney cells) and NIH-3T3 (embryonic cells) after exposure to NM-103 or NM-104 for 24h. Results are expressed as % INT light absorption means (± SE) of media control, cells without nanomaterials, which were set at 100%. Values were obtained from at least three independent experiments with four replicates each. Statistical analysis was performed using one way ANOVA followed by Tukey's *post hoc* test (*$p < 0.05$; **$p < 0.01$; ***$p < 0.001$). Necrosis of cells exposed to 0.01% Triton X-100 served as positive control for total cell lysis. Neither NM 103 nor NM 104 led to a significant increase of necrosis.

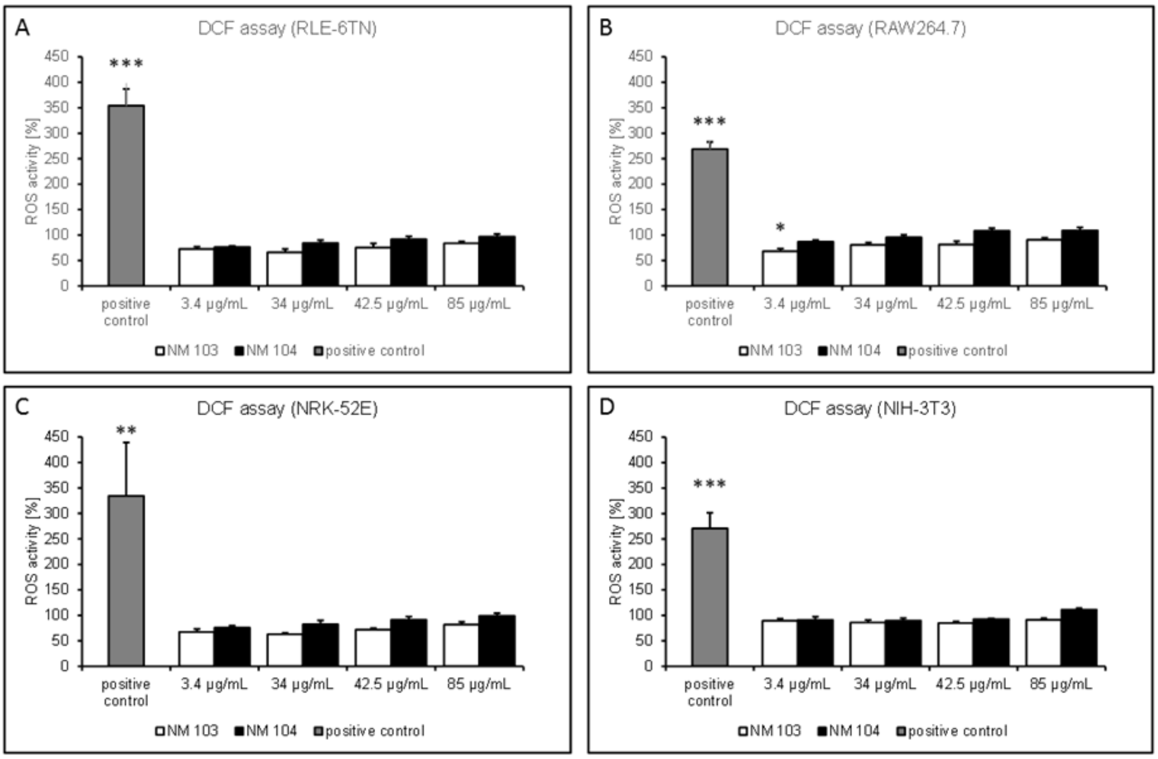

Fig. H. Effects of NM-103 and NM-104 on the intracellular concentration of reactive oxygen species (ROS) in of four different cell lines: RLE-6TN (A), RAW 264.7 (B), NRK-52E (C) and NIH-3T3 (D)

Results are expressed as % ROS activity of the medium control, cells without nanomaterials, and means (± SE) were obtained from at least four independent experiments with six replicates each. Statistical analysis was performed using one way ANOVA followed by Tukey's *post hoc* test (*$p < 0.05$; ** < 0.01; ***$p < 0.001$). Exposure to Carbon Black ENM (34 µg/ml equivalent of 10 µg/cm^2) served as a positive control. Neither NM-103 nor NM-104 led to a significant increase of cellular ROS activity, except for RAW 264.7 exposed to 3.4 µg/ml NM-103 (B) in which ROS was slightly decreased with low significance ($p < 0.05$).

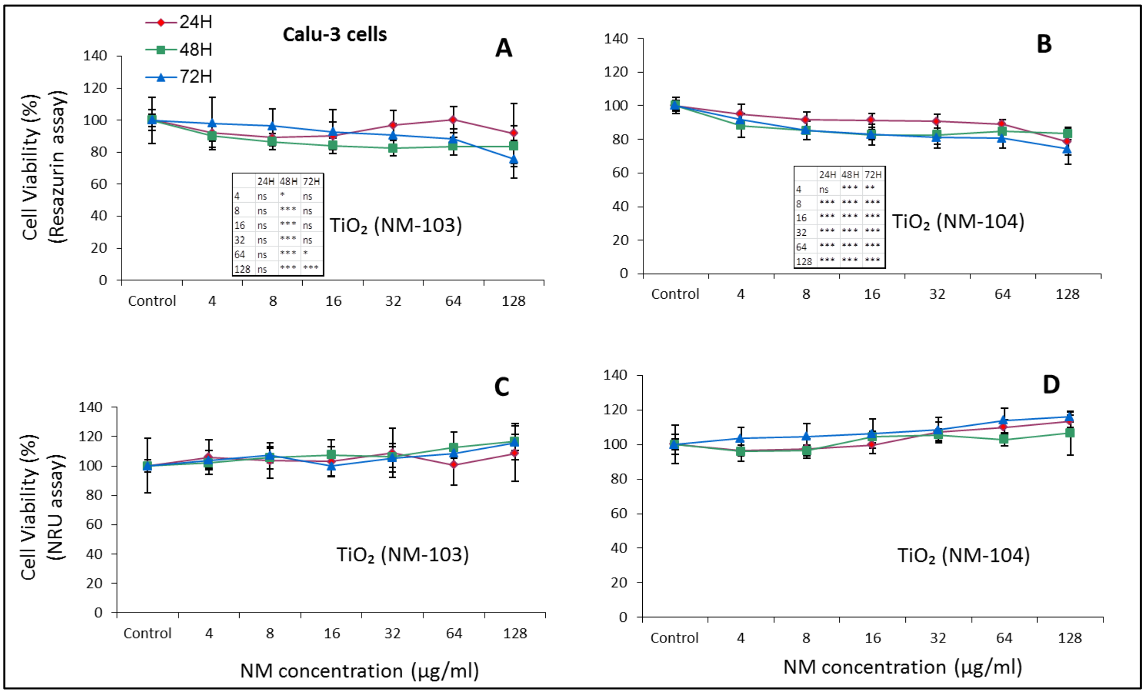

Fig. I. Calu-3 cells viability evaluated by Resazurin (A and B) and NRU (C and D) assays after exposure to TiO2 NM-103 and NM-104

Comparative evaluation of cell viability by resazurin assay (A and B) and NRU assay (C and D) after exposure to TiO2 (NM-103 and NM-104). Cells were grown for 24h in complete growth medium and then exposed for 24h, 48h and 72h to different concentrations of TiO2 NMs. At the end of the incubation, cell viability was assessed using two different assays (resazurin and NRU). Data are means ± SD of 10 independent determinations in two separate experiments. Statistical analysis was performed using one-way ANOVA followed by Bonferroni post-hoc test. *p< 0.05, ***p< 0.001.

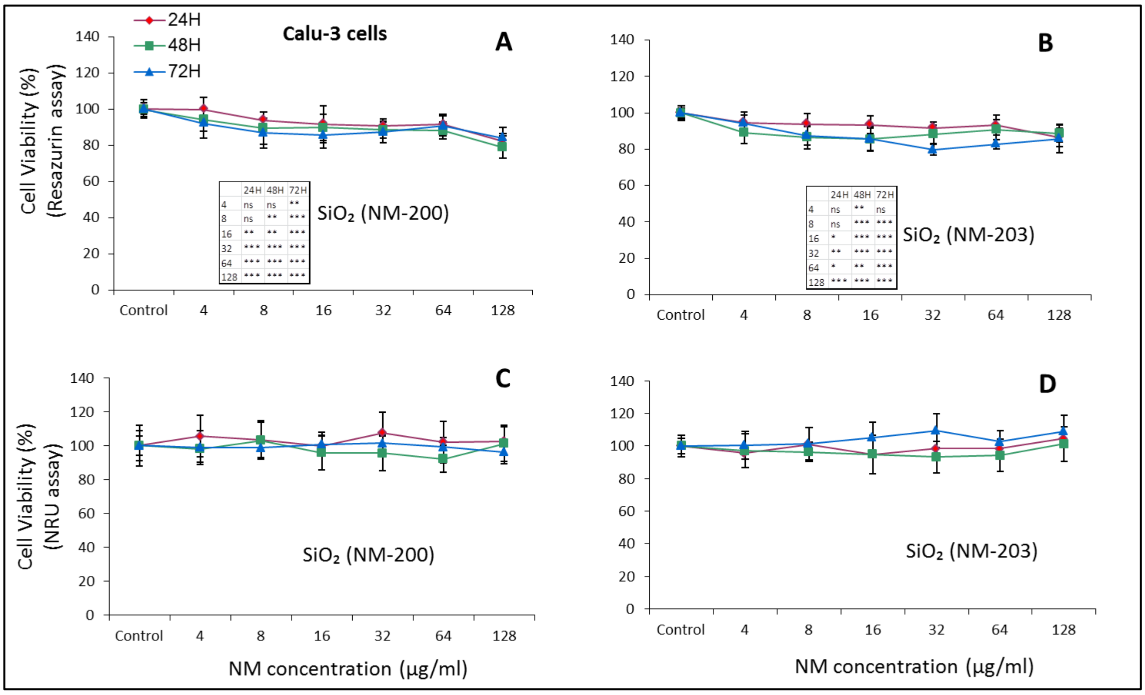

Fig. J. Calu-3 cells viability evaluated by Resazurin (A and B) and NRU (C and D) assays after exposure to SiO2 NM-200 and NM-203

Comparative evaluation of cell viability by resazurin assay (A and B) and NRU assay (C and D) after exposure to SiO2 (NM-200 and NM-203). Cells were grown for 24h in complete growth medium and then exposed for 24h, 48h and 72h to different concentrations of SiO2 NMs. At the end of the incubation, cell viability was assessed using two different assays (resazurin and NRU). Data are means ± SD of 10 independent determinations in two separate experiments. Statistical analysis was performed using one-way ANOVA followed by Bonferroni post-hoc test. $^{**}p < 0.01$, $^{***}p < 0.001$.

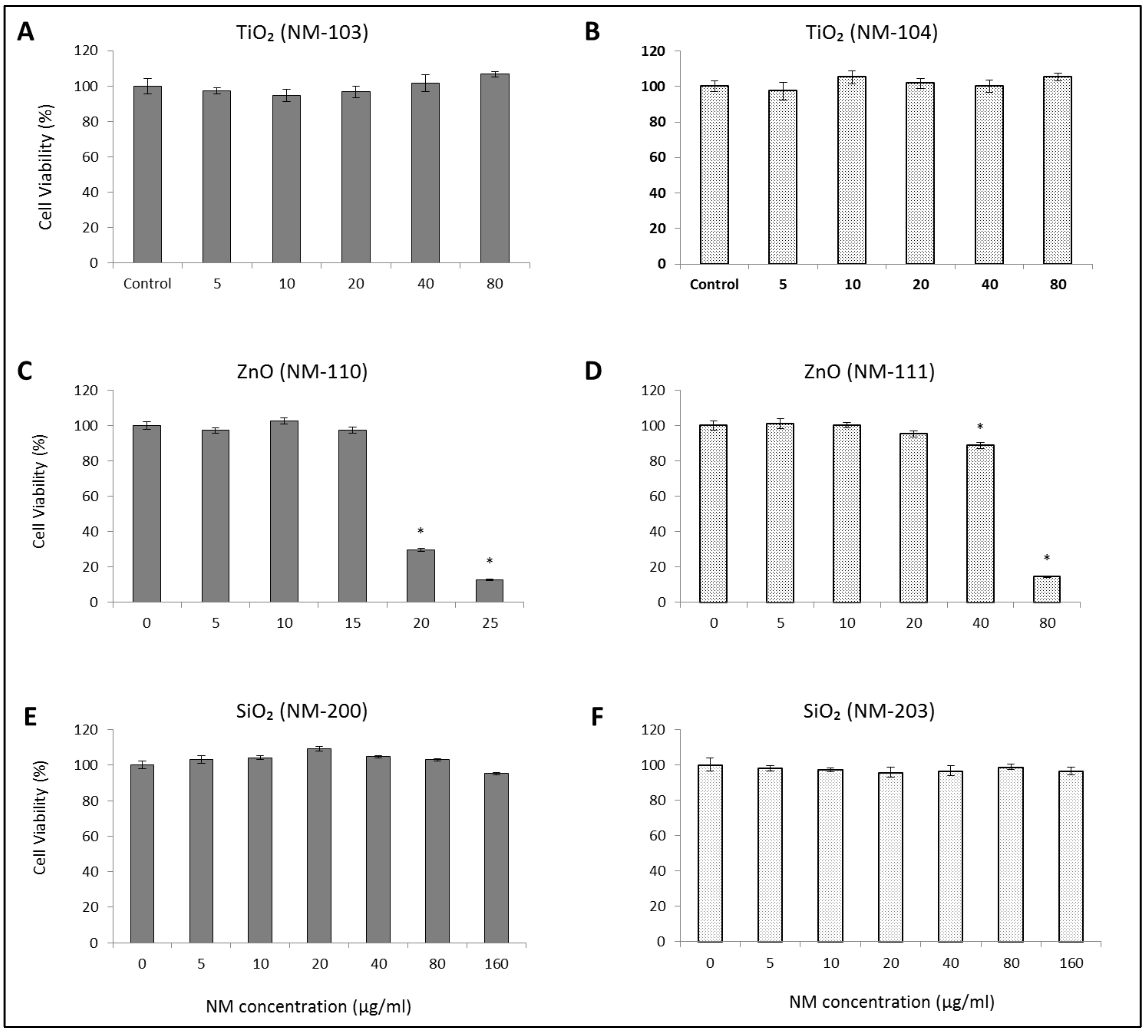

Fig. K. Cell viability of 16HBE after treatment for 24h with TiO_2 NM-103 (A) and NM-104 (B), ZnO NM-110 (C) and NM-111 (D) and SiO_2 NM-200 (E) and NM-203 (F)

Cell viability was determined by the CCK-8 assay kits (Dojindo Laboratories, Japan). After incubation with different concentrations of NMs for 24 h at 37° C, reagents were added according to the manufacturer's protocol. Absorbance was recorded at 450 nm by an Infinite M200 microplate reader (Tecan, Durham, USA). The mean absorbance of non-exposed cells was taken as the reference value (100% cellular viability). $*p < 0.05$, n = 5.

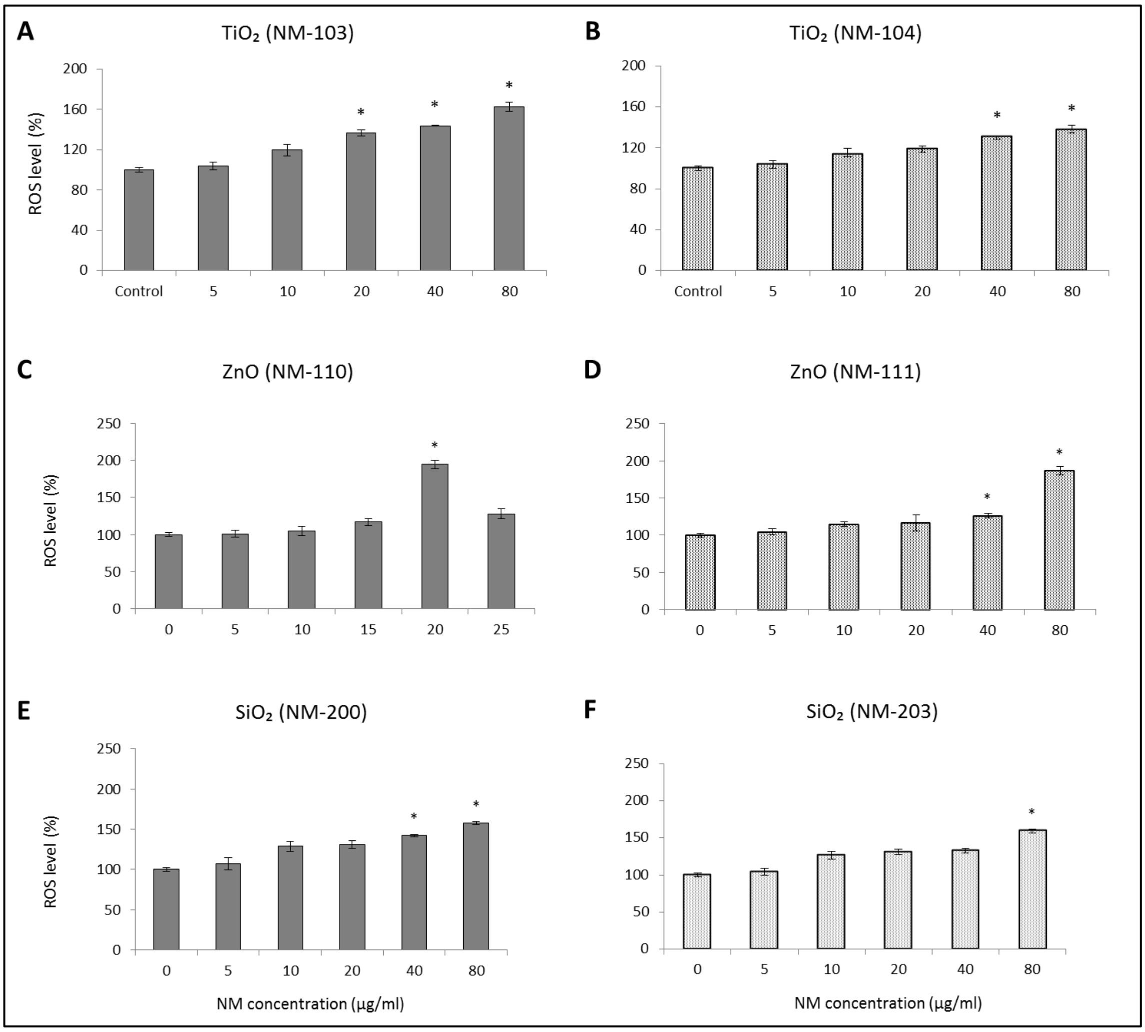

Fig. L. ROS level induced in 16HBE cells by TiO_2 NM-103 (A) and NM-104 (B), ZnO NM-110 (C) and NM-111 (D) and SiO_2 NM-200 (E) and NM-203 (F)

CM-H_2DCFDA and Hoechst 33342 were used to detect intracellular ROS and visualize nuclei after cells were treated for 24 hours, respectively. The average cellular florescence intensity (DCF staining) was expressed relative to the cell number after detection with IN cell analyzer 2000 (GE healthcare, USA). At least 1000 cells were analyzed in each treatment group ($*p<0.05$, experiments were repeated for three times).

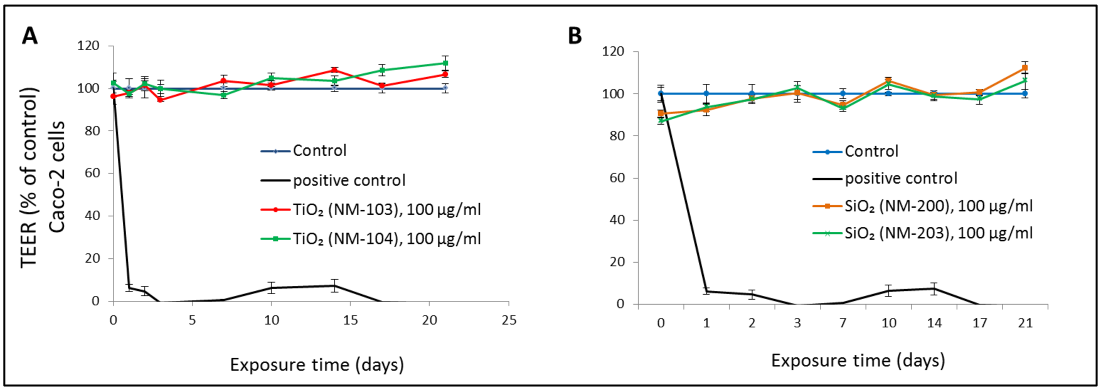

Fig. M. TEER measurements on Caco-2 epithelium during the exposure to TiO_2 NM-103 and NM-104 (A) and SiO_2 NM-200 and NM-203 (B)

The cells were exposed to repeated doses of NMs (day 1, 2, 3, 7, 10, 14 and 18) at a concentration of 100 µg/ml. After each change of treatment medium, the TEER was measured. TEER is reported as % of control (0.05% BSA).

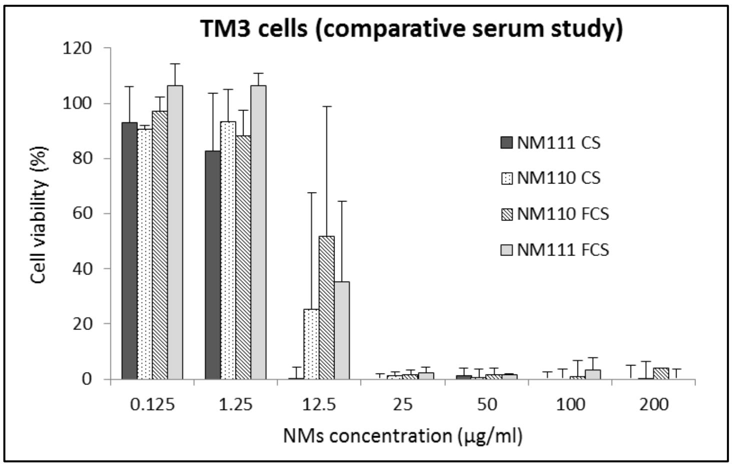

Fig. N. TM3 Cell viability following exposure to NM-110 & NM-111 using both standard and charcoal stripped FCS

WST-1 assay for cytotoxicity conducted for NM-110 (coated) and NM-111 (uncoated) forms of ZnO. All results were corrected for WST-1, as the control showed presence of interference in absorbance reading. At this stage within the work, a comparison between normal FCS and charcoal stripped FCS was undertaken (n=3). The result suggests that there is a clear effect on cell viability with increasing dose for either NMs. This was confirmed by one way ANOVA, which showed a significant decrease in viability from 12.5 µg/ml for NM-111 and 25 µg/ml for NM-110.
