# Supplementary material for: Comprehensive In Vitro Toxicity Testing of a Panel of Representative Oxide Nanomaterials: First Steps towards an Intelligent Testing Strategy
Source: PLoS One. 2015 May 21;10(5):e0127174. doi: 10.1371/journal.pone.0127174 (PMC4440714; doi:10.1371/journal.pone.0127174)
Supplement: S3 File — (PDF) [file pone.0127174.s003.pdf]

Supporting Information – S3 File

Evaluation of oxide nanomaterials interferences with LDH, ELISA, Resazurin, NRU and WST-8 and WST-1 assays

TiO_2 NMs

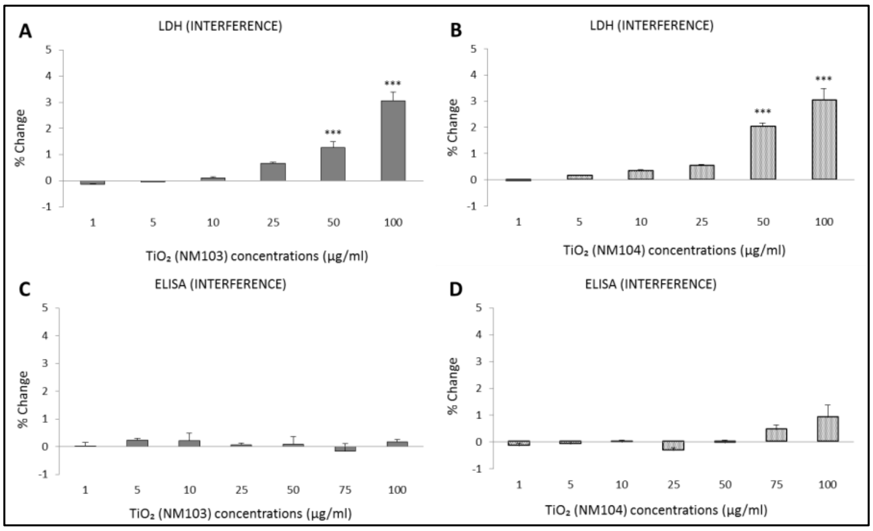

Fig. O. Interference of Titanium dioxide nanomaterials (NM-103 and NM-104) with LDH assay (A and B) and ELISA (C and D)

A significant interference with the LDH assay in a dose-dependent manner was observed. However at the doses used in our studies the maximum percent change induced by both nanomaterials was around 3%. Values of interference with the ELISA were always below 1%.

For WST-1 assay (*data not shown*), both NM-103 and NM-104, control experiment wells containing NMs and cells only were shown to have minimal absorbance readings, thus it was concluded that there was no interference from TiO2 on the assay.

For WST-8 assay the interferences with TiO_2 used for the cytotoxicity testing shown in Figures S6-S8 in File S3 were performed as described previously (*Kroll et al. 2012*). The used concentrations were below the observed interfering concentrations.

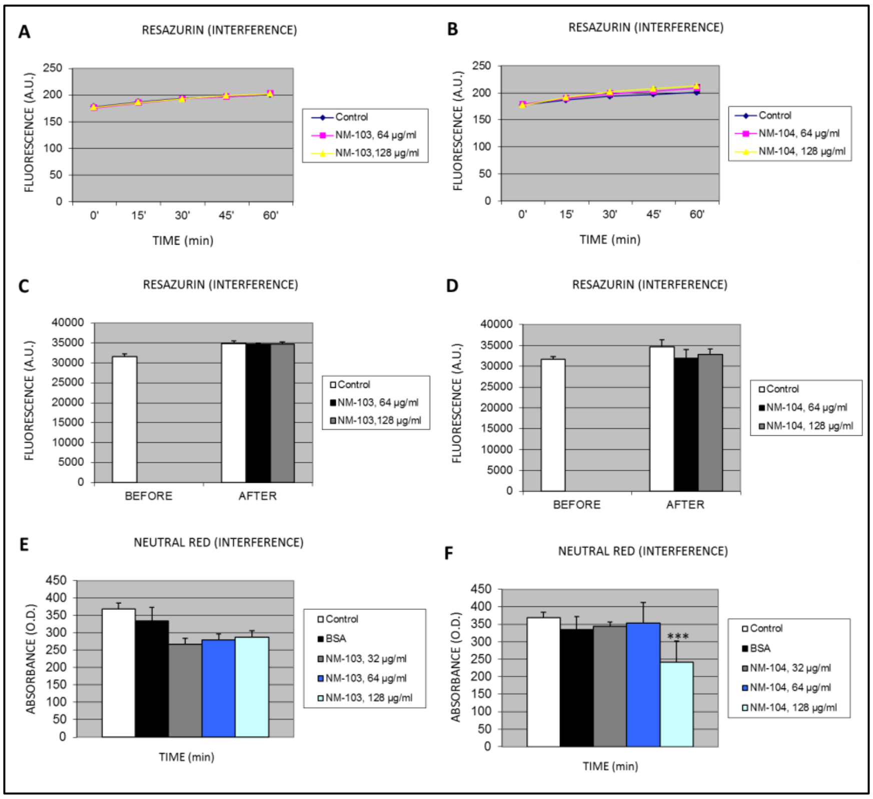

Fig. P. Interferences of TiO_2 (NM-103 and NM-104) with resazurin and NRU assays

The fluorescence read at the 60 min time point in the absence of cells was not significantly influenced by TiO_2 NMs and < 0.5% of the minimal value obtained in the presence of cells. It is concluded that TiO_2 NMs do not interfere with the resazurin method through autofluorescence (A and B). The supplementation of medium with TiO_2 NMs did not appreciably quench resazurin-derived fluorescence (C and D). For the NRU assay (E and F), the results indicate that addition of neutral red solution with TiO_2 (NM-103 or NM-104). NMs decrease OD values and showing a binding of neutral red to the NMs. However, binding is not dose dependent, suggesting that only a fraction of the dye solution (which is not homogeneous) is adsorbed to the NMs. Neutral Red binding to TiO_2 NMs should remove the dye to the solution and, hence, lower its uptake by the cells, factitiously lowering viability signal. Alternatively, given that NMs enter cells, dye binding could increase its accumulation in the cells and, hence, increase viability signal. Actually, results of neutral red assay indicate smaller viability decreases compared with the resazurin assay. Data are means ± SD of 6 independent determinations in two separate experiments. Statistical analysis was performed using one-way ANOVA followed by Bonferroni post-hoc test. *$p < 0.05$, ***$p < 0.001$.

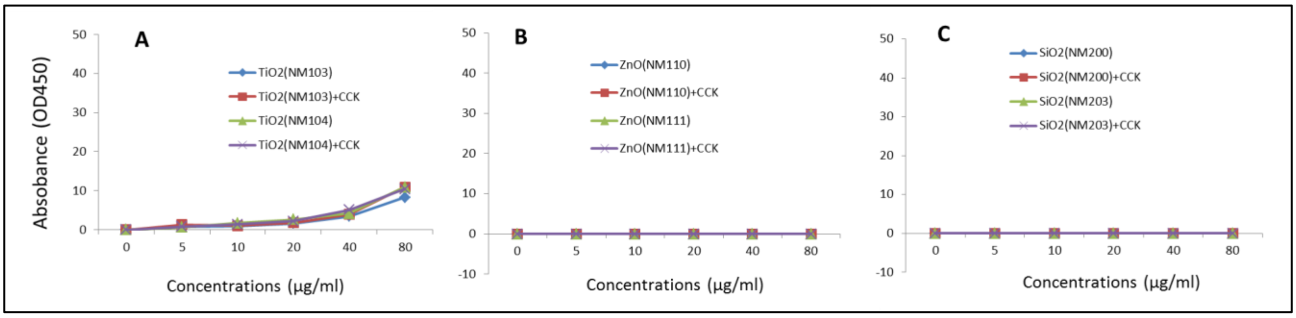

Fig. Q. Interferences the six NMs tested with the WST-8 (CCK) assay. Interferences with the assay readouts were observed in the case of both TiO_2 NMs (A) but not for ZnO and SiO_2 (B and C)

In the estimation of interference of WST-8 (CCK) assay, NMs were directly added to the test system without cells. The absorbance of OD450 was measured with or without CCK solution stain. The difference between the regular test procedures to this no-cell WST-8 test assay was that most NMs in the incubation medium being washed away before CCK solution added in the regular ones. There was only about 10 percent of the cell viability over-estimation at 80 μg/ml TiO_2 treatment condition in no-cell test, and even much lower at other lower exposure concentrations. Therefore, the actual test interference of those residue NMs was quite low and not likely to influence the results significantly.

ZnO NMs

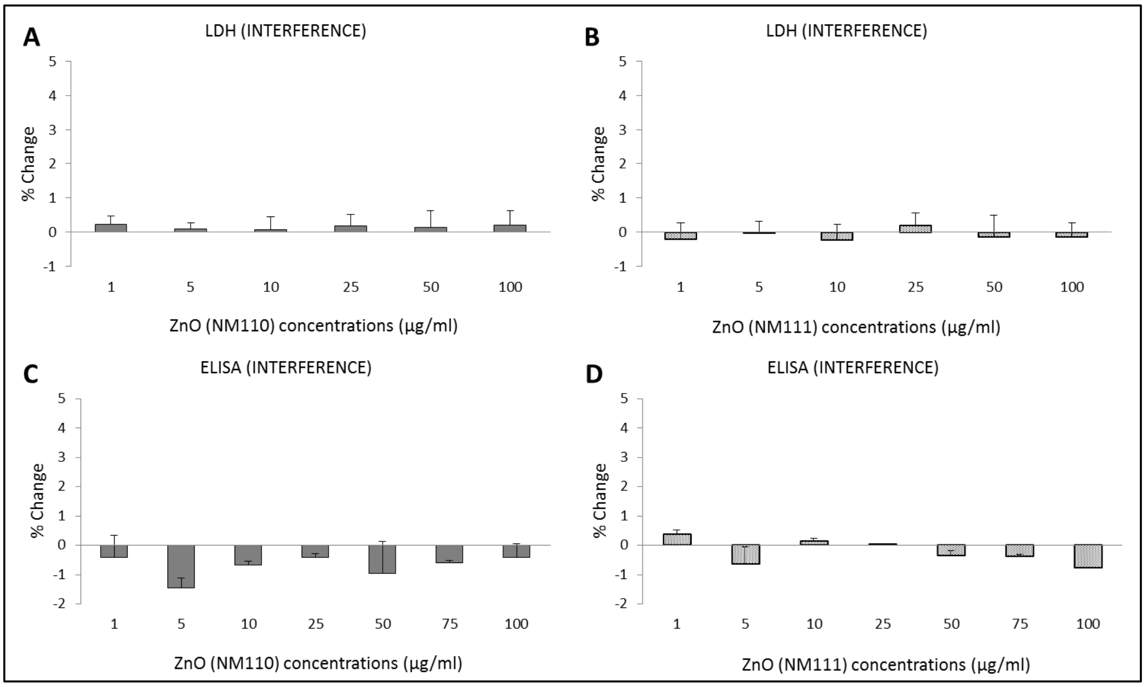

Fig. R. Interference of Zinc oxide nanomaterials (NM-110 and NM-111) with LDH assay (A and B) and ELISA (C and D)

The results showed that NM-110 and NM-111 do not interfere with the LDH assay as the values of interference were always below 0.25%. The evaluation of NMs-assay interferences showed that none of the ZnO NMs interferes with the ELISA assay.

For the WST-1 assay (*data not shown*), both NM-110 and NM-111 shown to have minimal absorbance readings, thus it was concluded that there was no interference from ZnO in this assay.

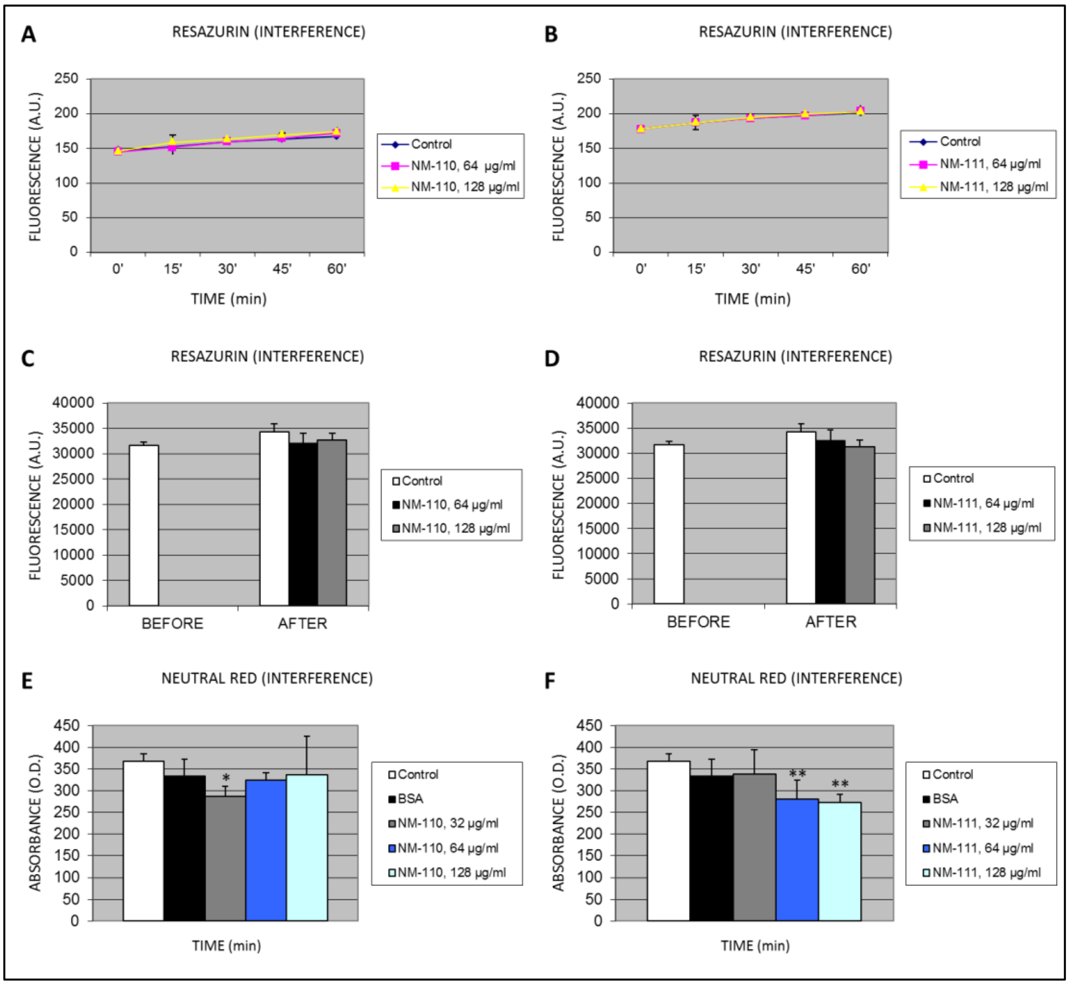

Fig. S. Interferences of ZnO (NM-110 and NM-111) with resazurin and NRU

The fluorescence read at the 60 min time point in the absence of cells was not significantly influenced by ZnO NMs and < 0.5% of the minimal value obtained in the presence of cells. It is concluded that ZnO NMs do not interfere with the resazurin method through autofluorescence (A and B). The supplementation of medium with ZnO NMs did not appreciably quench resazurin-derived fluorescence (C and D). For the NRU assay (E and F), the results indicate that addition of neutral red solution with ZnO (NM-110 or NM-111). NMs decrease OD values and showing a binding of neutral red to the NMs. However, binding is not dose dependent, suggesting that only a fraction of the dye solution (which is not homogeneous) is adsorbed to the NMs. Neutral Red binding to ZnO NMs apparently decreases the free dye concentration in the solution and, hence, lowers its uptake by the cells, factitiously lowering viability signal. Alternatively, given that NMs enter cells, dye binding could increase its accumulation in the cells and, hence, increase viability

signal. Actually, results of neutral red assay indicate smaller viability decreases compared with the resazurin assay. Data are means ± SD of 10 independent determinations in two separate experiments. Statistical analysis was performed using one-way ANOVA followed by Bonferroni post-hoc test. *$p < 0.05$, **$p < 0.01$.

SiO_2 NMs

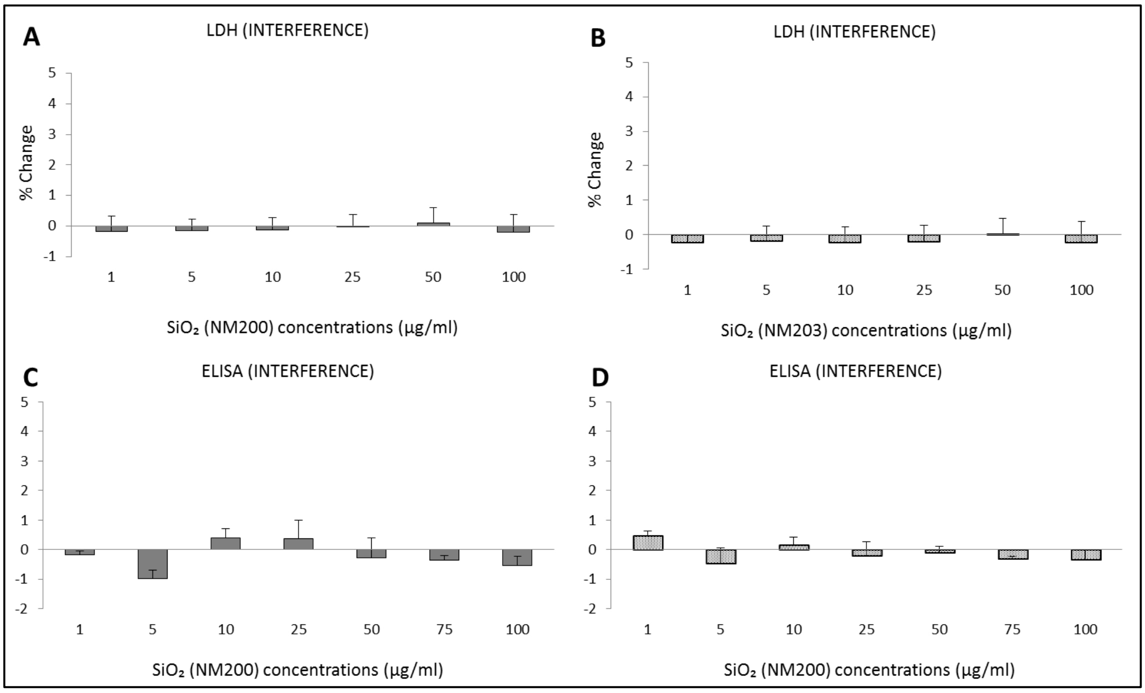

Fig. T. Interference evaluation of SiO2 (NM-200 and NM-203) with LDH assay (A and B) and ELISA (C and D)

The results showed that both NMs (NM-200 and NM-203) do not interfere with the LDH assay as the values of interference were always below 0,25%. Also none of the SiO2 NMs interferes with the ELISA.

WST-1 assay (*data not shown*) - for both NM-200 and NM-203, control wells containing NM and wells containing cells only were shown to have minimal absorbance readings, thus it was concluded that there was no interference from the SiO_2.

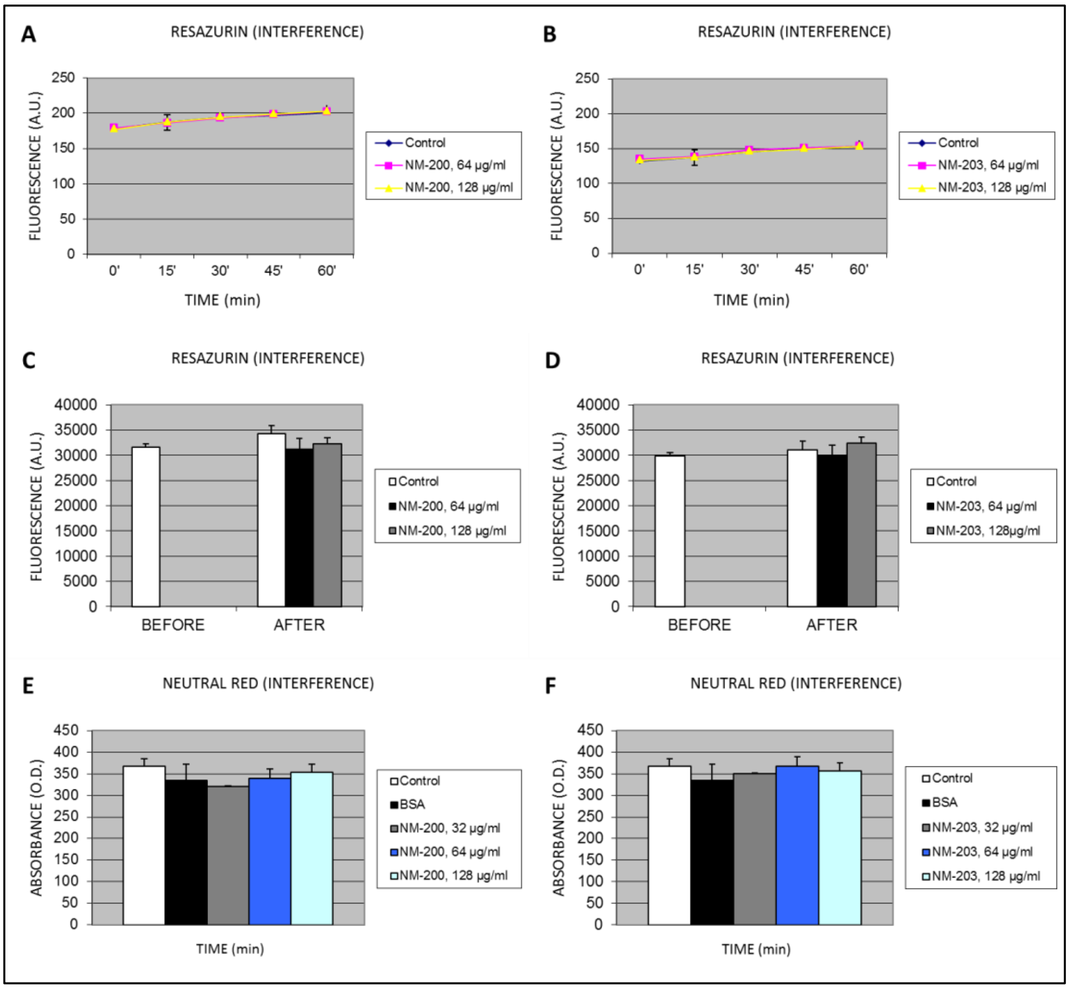

Fig. U. Interferences of SiO_2 NMs with resazurin (A-D) and NRU (E-F) assays

The fluorescence read at the 60 min time point was not significantly influenced by SiO2 NMs and < 0.5% of the minimal value obtained in the presence of cells. It is concluded that SiO_2 NMs do not interfere with the resazurin method through autofluorescence (A and B) while the addition of SiO_2 NMs in culture medium did not appreciably quench resazurin-derived fluorescence (C and D). Regarding the assay-NMs interferences with NRU assay (E and F), the results indicate that the supplementation of neutral red solution with SiO_2 NMs did not influence the OD values, indicating the absence of significant dye binding. Data are means ± SD of 10 independent determinations in two separate experiments. Statistical analysis was performed using one-way ANOVA followed by Bonferroni post-hoc test.
